# The Diversity of Plastisphere Bacterial and Fungal Communities Differs between Biodegradable Polymer Types in Soil

**DOI:** 10.1007/s00248-025-02677-z

**Published:** 2026-02-03

**Authors:** Rebecca Lyons, Clement M. Chan, Catherine M. E. Hodal, Andrew R. Parry, Paul Lant, Steven Pratt, Bronwyn Laycock, Paul G. Dennis

**Affiliations:** 1 School of the Environment, The University of Queensland, St Lucia, QLD Australia; 2 School of Chemical Engineering, The University of Queensland, St Lucia, QLD Australia

**Keywords:** Poly(3-hydroxybutyrate-co-3-hydroxyvalerate) (PHBV), Poly(butylene adipate-*co*-terephthalate) (PBAT), Poly(butylene succinate) (PBS), Poly(lactic acid) (PLA), Biodegradation in soil, Microbial communities, biodegradable polymers

## Abstract

Biodegradable polymers (BPs) are often seen as sustainable alternatives to conventional plastics. However, their actual degradability in soils, a major sink for plastic pollution, varies considerably. Moreover, their breakdown can influence soil microbial communities, with potential consequences for ecosystem function. Here, we compared the impacts of four major BP types: (1) poly(3-hydroxybutyrate-*co*-3-hydroxyvalerate) (PHBV), (2) poly(butylene adipate-*co*-terephthalate) (PBAT), (3) poly(butylene succinate) (PBS), and (4) poly(lactic acid) (PLA), on soil and plastisphere microbial communities. Standardised BP samples were buried in soil for 117 days, after which bacterial and fungal biomass load and diversity were assessed in the BPs and surrounding soils using quantitative PCR and metabarcoding. X-ray micro-computed tomography (µXCT) and gel permeation chromatography (GPC) were also used to compare signs of degradation. PLA exhibited negligible signs of degradation, no evidence of microbial colonisation, and minimal impacts on microbial diversity relative to bulk soil. In contrast, PHBV, PBAT, and PBS underwent marked degradation and supported distinct microbial assemblages with elevated biomass but reduced diversity, indicating selective enrichment of specific taxa. These findings demonstrate that BPs differ markedly in their environmental degradability and influence on soil microbial communities, providing insights to support polymer selection for more sustainable plastic use.

**Supplementary Information:**

The online version contains supplementary material available at 10.1007/s00248-025-02677-z.

## Introduction

To reduce the accumulation of plastics in the environment, the use and manufacture of biodegradable plastics is increasing [1, 2]. By 2028, the majority (> 90%) of global biodegradable plastic production is predicted to be dominated by: (1) poly(lactic acid) (PLA; 70%); (2) the polyhydroxyalkanoates (PHAs; 22%), (3) poly(butylene adipate-*co*-terephthalate) (PBAT; 2%) and (4) poly(butylene succinate) (PBS; 0.4%) [2]. As the use of plastic products containing these biodegradable polymers (BPs) increases, their leakage into terrestrial ecosystems is inevitable making soils a major sink [3, 4]. Within soils, PHAs, PBAT, PBS and PLA have been shown to degrade to different extents and at different rates, with copolymer content, material processing, the presence of additives, and environmental conditions influencing biodegradation processes [5–7]. For example, PHBV (poly(3-hydroxybutyrate-*co*−3-hydroxyvalerate) – a common PHA) is readily degradable in soil [8], whereas PLA may persist much longer in soil under ambient conditions [9]. A recent study comparing PBS, PHBV and PLA buried in soil for 112 days found complete disintegration of PBS, partial degradation of PHBV, and no visible signs of degradation for PLA [10]. For the latter, these findings are consistent with predictions that it may take years to degrade in soil [2]. Some studies, though, report significant degradation and disintegration of PLA in soil after approximately half a year [11, 12]. Nonetheless, products marketed as being biodegradable may degrade at rates that are unacceptably slow within the environments that they are likely to contaminate, ultimately contributing to plastic pollution [13, 14].

A further consideration when evaluating the suitability of BPs for products likely to enter soils is whether their degradation is associated with significant ecological impacts [15]. In soil, distinct microbial communities have been shown to form on the surfaces of PHBV, PBAT, PBS and PLA (i.e., the plastisphere) relative to those in surrounding bulk soil, altering the diversity and composition of these communities [16–18]. As soil microbiomes mediate important soil ecosystem services, changes in their diversity may influence plant and soil health [19]. Hence, further work is required to ascertain the sustainability credentials of these BPs, even though they are at least partially degradable in soils [20, 21]. It is important to acknowledge that real plastic products contain not only BPs but other additives that may influence microbial attachment, surface hydrophobicity, and degradation kinetics. However, focussing on pure BPs provides a reproducible baseline to understand how different types of polymer influence soil microbial communities, without the confounding effects of other plastic constituents. While some studies have investigated the effects of pure BPs on soil microbial communities [16, 18, 22] it is unclear how their effects compare with one another.

Here, we buried samples of PHBV, PBAT, PBS and PLA in soil for 117 days and then characterised: (1) a range of physicochemical and molecular changes that occurred throughout the incubation period, and (2) the diversity and ‘biomass’ of bacterial and fungal communities. All experiments were performed using the ISO 17556:2019 global standard soil for determining plastic biodegradability in soils [23]. We choose this approach to enable more reproducible comparisons of polymer behaviour between studies and minimise the influence of localised edaphic properties. At the end of the experiment, DNA was extracted from BP pieces, soil attached to the BPs, and the bulk soil. The diversity and composition of bacterial and fungal communities, as well as their ‘biomass’ load, were analysed using phylogenetic marker gene sequencing and quantitative PCR (qPCR), respectively. We assessed the impact of soil burial on visual and physical properties using X-ray micro-computed tomography (µXCT) as well as molecular weight changes using gel permeation chromatography (GPC). We hypothesised that PBS would exhibit the strongest signs of degradation and PLA the least. Furthermore, we hypothesised that the diversity and load of bacterial and fungal communities would differ between BP types and that the impacts of BPs would diminish from the polymer surface to the bulk soil.

## Materials and methods

### Preparation of Biodegradable Polymer Samples

A co-rotating twin screw extruder with a diameter of 16 mm and a length-to-diameter ratio of 40:1 was used for producing melt-processed BP sheets. BP materials (Table S1) were first dried in a vacuum oven at 60 °C and a gauge pressure of −80 kPa overnight. The powders or pellets were fed to the extruder with a spring feeder. A decreasing temperature profile with a maximum barrel temperature of 180 °C and a die temperature of 160 °C was implemented (Table S2). The screw speed was maintained at 100 rpm. The screw configuration was chosen to consist of kneading elements to provide moderate mixing. A slit die with a cross-section dimension of 20 × 2 mm was placed at the die to yield sections of rectangular strips. Strips were cut using a sterilised razor blade to 20 mm x 20 mm x 2 mm, stored in a sterile (UV-treated) plastic ziplock bags and handled with sterilised tweezers for biodegradation tests.

### Experimental Design

A standardised soil batch with a moisture content of 60% soil water holding capacity was prepared according to ISO 17556:2019 [23] as previously described [24]. Each jar was randomly assigned a BP type (PHBV, PBAT, PLA, PBS), so that there were five independent biological replicates per BP type. For each replicate, 70 g of soil was layered in a 100 mL glass jar, with 17.5 g of soil per layer and a BP piece (20 mm × 20 mm × 2 mm) placed between each layer. The jars were loosely sealed to allow gas exchange, thereby minimizing oxygen limitation and CO₂ accumulation, while limiting excessive moisture loss, and were incubated (in a randomized design on the same shelf) at 25 °C for 117 days. Moisture content was monitored and maintained weekly by weighing the jars and replenishing any lost moisture with sterile distilled water.

#### Sample collection

**Fig. 1:**
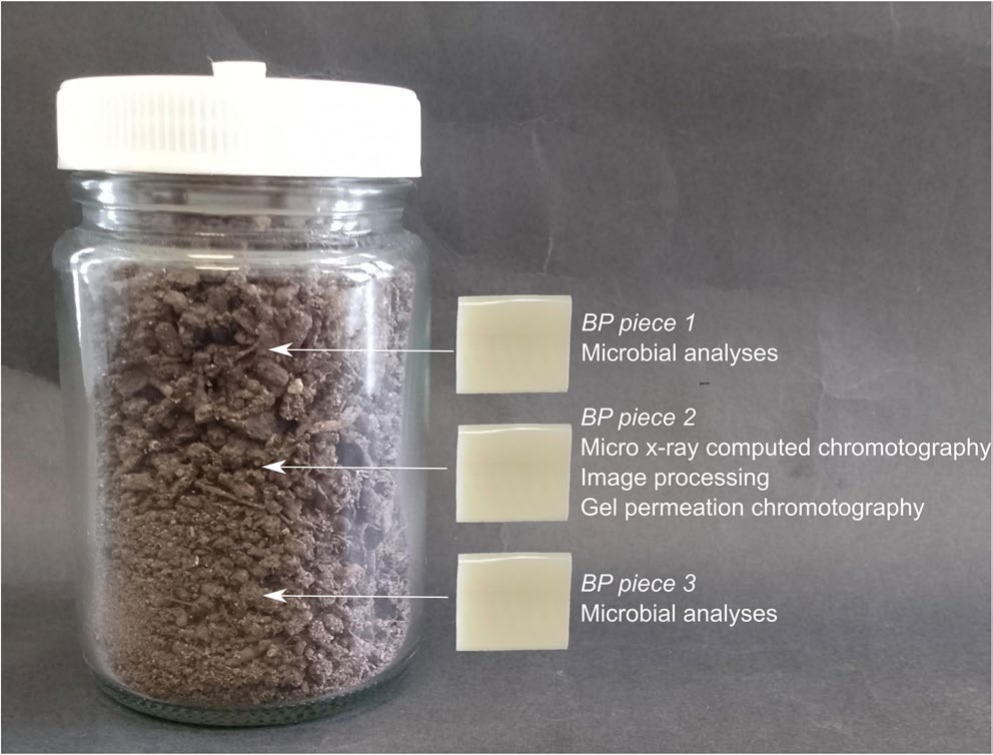
Experimental design showing the arrangement of one biological replicate (jar). Arrows show the location of the buried BP pieces, which were placed horizontally in the jar

On day 117 of burial, BP piece 2 (Fig. 1) was retrieved using sterile forceps and rinsed with sterile distilled water to remove any adhering soil. These samples were photographed and analysed alongside non-buried (0 day) controls using µXCT and GPC. For microbial analyses, BP pieces 1 and 3 were collected, and the soil attached to each piece was carefully scraped off using a sterile scalpel blade. While care was taken to remove as much soil as possible, minor residues remained adhered to the BP surface. The attached soils from BP pieces 1 and 3 from each jar were then pooled to create one composite ‘attached soil’ sample, while the BP pieces themselves were also combined into one composite ‘polymer’ sample. The remaining bulk soil in each jar was then collected. The three compartments designated for microbial analysis were therefore ‘bulk soil’, ‘attached soil’, and ‘polymer’. All samples for microbial analyses were stored at −80°C, freeze-dried, and then ground and homogenized using a TissueLyser II (Qiagen) at 30 Hz for 5 min. For the polymer compartment samples, samples were ground to as fine a consistency as possible, however as some small BP segments remained, the entire samples were used for the DNA extraction.

#### Physicochemical and visual characterization of BP samples

 To supplement photographs documenting visual signs of degradation in buried (117 days) and non-buried (0 days) BP samples, we used µXCT to assess physical integrity and GPC to determine the molecular weight of buried and non-buried BP pieces, as previously described [25]; full details are provided in our Supplementary Methods.

### Bacterial and Fungal Biomass and Diversity

#### DNA extraction

For soil samples, 250 mg pre-ground lyophilized soil was added to 1 mL extraction buffer (0.12 M Na_2_HPO_4_, 1% SDS). For each polymer sample, the entire pre-ground lyophilized piece ranging from 1530 to 1950 mg dry weight was added to 1.5 mL extraction buffer. This ensured that we captured as much biomass from the polymer as possible and helped reduced any bias associated with heterogenous colonisation. Samples were lysed in buffer using a TissueLyser II (Qiagen) at 30 Hz for 3 min. The homogenate was centrifuged at 16,000 rcf for 5 min to remove debris and the supernatant was extracted with phenol: chloroform: isoamyl alcohol (IAA), followed by chloroform: IAA. DNA was precipitated with sodium acetate and isopropanol and then centrifuged at 16,000 rcf for 5 min. The DNA pellet was rinsed with 75% ethanol and resuspended in 100 µL molecular biology grade water. To remove inhibitors, the resuspended DNA was purified using the QIAquick Gel Extraction Kit (Qiagen) according to the manufacturer’s instructions and eluted in 50 µL of molecular biology grade water.

#### Real-time PCR quantification of bacterial and fungal marker gene copies

Quantitative PCR (qPCR) assays targeting bacterial (16 S) and fungal (ITS) marker genes were performed in triplicate for each sample using universal primer pairs 338-Eub/513Eub and ITS1f/5.8s (Table S3), respectively, as previously described [24]. Full details are provided in our Supplementary Methods.

#### Phylogenetic marker gene sequencing

16 S and ITS2 rRNA genes were amplified using universal primer pairs 926 F/1392wR and gITS7F/ITS4R respectively, modified to contain Illumina-specific adapter sequences and custom molecular identifiers (Table S3). Full details are provided in our Supplementary Methods. Libraries were sequenced on an Illumina MiSeq using 30% PhiX Control v3 (Illumina) and a MiSeq Reagent Kit v3 (600 cycles; Illumina) according to the manufacturer’s instructions. Insufficient 16 S rRNA gene reads were obtained for the PBAT polymer compartment sample from replicate (jar) 1, so this sample was excluded from the metabarcoding analyses.

#### Processing of sequence data

A modified UPARSE pipeline [26] was used to process the sequences. Forward reads were: (1) demultiplexed with *cutadapt* in QIIME2 (v2017.9.0 [27, 28]); (2) quality filtered using USEARCH (v10.0.240 [29]); and then (3) mapped against representative sequences using *fastx_uniques* and *cluster_otus* (sequence similarity = 0.97) in USEARCH to create OTU tables. Prior to clustering, fungal ITS2 regions were extracted with ITSx (v1.0.11 [30]) and chimeras were removed using uchime2_ref of USEARCH against the UNITE v10.0 database [31]. Taxonomy was assigned to bacterial OTUs using Greengenes2 (v 2022.10) [32] and to fungal OTUs using UNITE (v10.0) via BLASTN (v2.3.0+ [33]) within the QIIME2 feature classifier (v2017.9 [28]). OTUs classified as chloroplasts, mitochondria, archaea, or eukaryotes were filtered out using the BIOM tool suite [34]. OTU tables were then rarefied to 5,000 (16 S) or 5,850 (ITS2) sequences per sample and the following alpha diversity metrics were calculated: observed numbers of OTUs (S_obs_), predicted richness (Chao1 [35]), and Shannon’s Diversity [36] Index. For bacterial communities, MAFFT (v7.221 [37]) was used to align representative 16 S OTU sequences and then Fast-Tree (v2.1.9 [38]) was used to generating a midpoint-rooted phylogenetic tree. Faith’s Phylogenetic Diversity (PD [39]) and Weighted UniFrac distances [40] were then calculated from the tree to represent phylogenetic metrics of alpha and beta diversity, respectively.

### Statistical Analyses

#### Physicochemical characteristics

Two-tailed student’s t-tests were used to determine whether the number average molecular weight ($\:{\stackrel{-}{M}}_{n}$), weight average molecular weight ($\:{\stackrel{-}{M}}_{W}$) or dispersity (Đ) differed significantly between non-buried (0 days) and buried (117 days) BP samples. To measure the percentage change for each variable in buried vs. non-buried samples, the value for each buried biological replicate was divided by the mean of the five non-buried (0 days) BP replicates and the difference was converted to percentage change. One-way ANOVAs were then used to determine the effect of BP type (PHBV, PBAT, PBS, PLA) on the percentage change in each variable ($\:{\stackrel{-}{M}}_{n}$, $\:{\stackrel{-}{M}}_{W}$, Đ). Shapiro-Wilk and Levene’s tests were used to assess and confirm the normality of residuals and homoscedasticity, respectively (Table S4). Tukey’s *post hoc* analyses were then performed on significant models.

#### Biomass load and alpha diversity of bacterial and fungal communities

To assess the fixed main and interactive effects BP type and compartment on the alpha diversity and biomass of microbial communities we used generalized linear mixed models (GLMM) with a Gamma (log link) distribution and jar as a random effect (to account for the repeated measures and potential jar effects). GLMM with a Gamma (log link) distribution was deemed appropriate as Shapiro-Wilk and Levene’s tests indicated that the assumptions for LMM were not adequately met irrespective of whether the response data were raw or log_10_ transformed (Table S4). GLMMs and Type III Wald χ² tests were performed using the ‘*glmmTMB’* [41] and ‘*car’* [42] *R* packages, respectively. Post-hoc pairwise comparisons of estimated marginal means were conducted using the ‘*emmeans’* package [43] with *p* values adjusted for multiple testing using the Šidák correction. The qPCR counts expressed per cm^− 2^ (measured for the polymer compartment only) were log_10_-transformed and then subjected to one-way ANOVA with BP type as the predictor and then followed with a Tukey post-hoc test.

#### Beta diversity of bacterial and fungal communities

Permutational multivariate analysis of variance (PERMANOVA) was used to identify the main and interactive effects of ‘BP type’ (PHBV, PBAT, PBS, PLA) and ‘compartment’ (bulk soil, attached soil, polymer) on Hellinger-transformed OTU relative abundances [44] and weighted UniFrac distances [45]. PERMANOVA was implemented using vegan::adonis2 [46]. Distance-based redundancy analyses (db-RDA) constrained by BP type and compartment were used to visualize the compositional similarities between samples. These db-RDA ordinations were based on Hellinger-transformed OTU relative abundances and were generated using the function vegan::rda [46]. We also used indicator analysis using the function labdsv::indval [47] to identify OTUs that were significant indictors of different treatments. These analyses were also performed on Hellinger-transformed OTU abundances, and only OTUs with a relative abundance ≥ 0.5% in at least one sample were included.

## Results

### Physicochemical and Visual Markers of BP Degradation

**Fig. 2:**
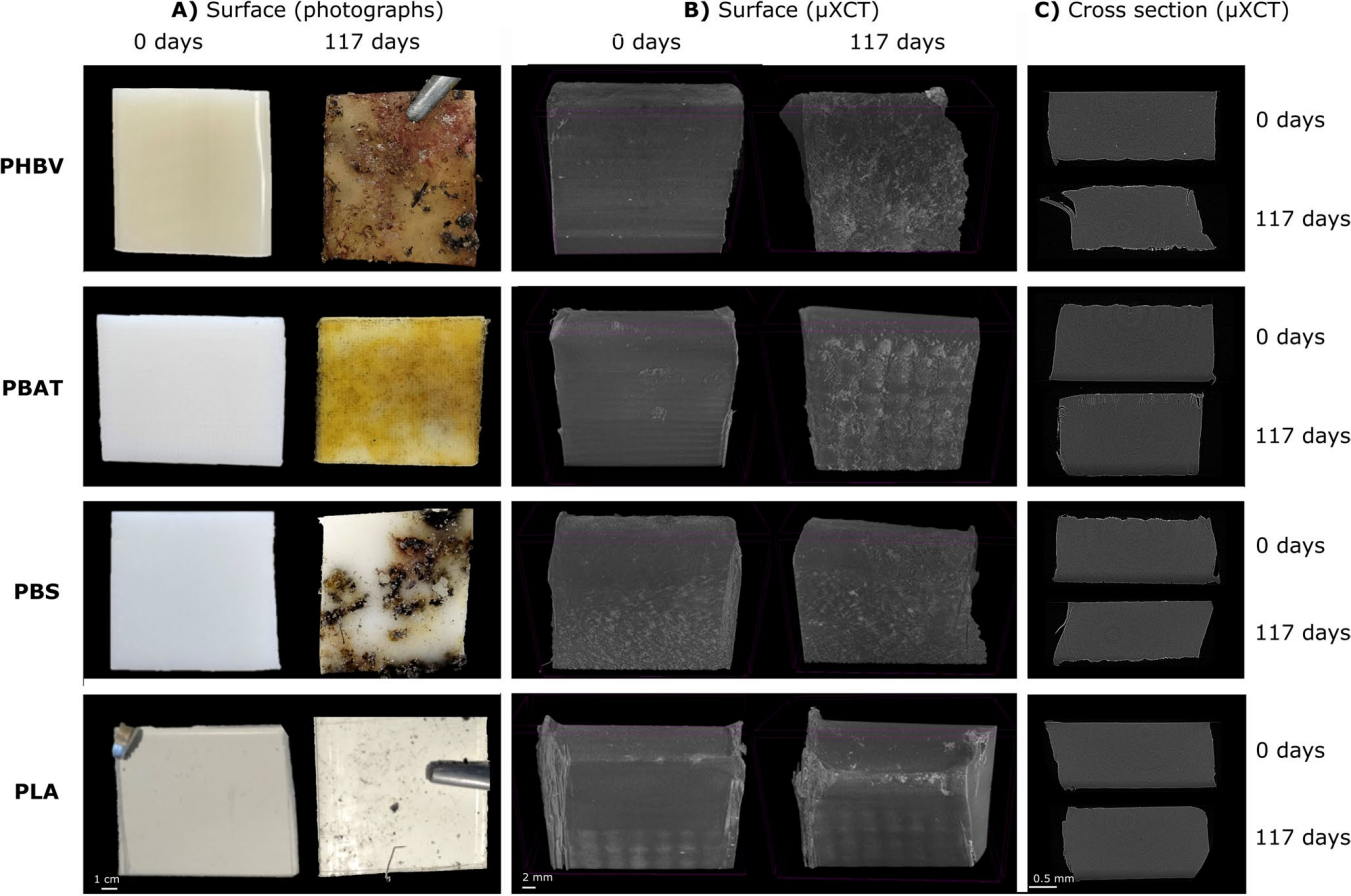
Visual inspection of non-incubated (0 days) and incubated (117 days) biodegradable polymers (BPs). BP pieces were visualised using (**A**) photography and (**B**, **C**) X-ray micro computed tomography (µXCT). Cross section images (**C**) are the composite images of scans through 2 mm thick pieces of BP

Visual inspection and µXCT analysis revealed surface deterioration and discoloration in all buried BPs compared to their non-buried counterparts (Fig. 2). Relative to the non-buried samples: (1) buried PHBV exhibited complete surface discoloration, increased edge erosion, and surface roughness, (2) buried PBAT exhibited complete surface discoloration and increased surface roughness with cracking extending to approximately one-fifth of the total thickness of the sample with minimal edge erosion, and (3) PBS exhibited partial, patchy surface discoloration with increased edge erosion (Fig. 2). Relative to the other BPs, PLA exhibited minor changes in surface colour, with negligible edge erosion and roughness alterations (Fig. 2). Consistent with the deeper cracks observed in PBAT, of the four BPs, PBAT showed the greatest increases in porosity following soil burial (Table S5).

**Fig. 3:**
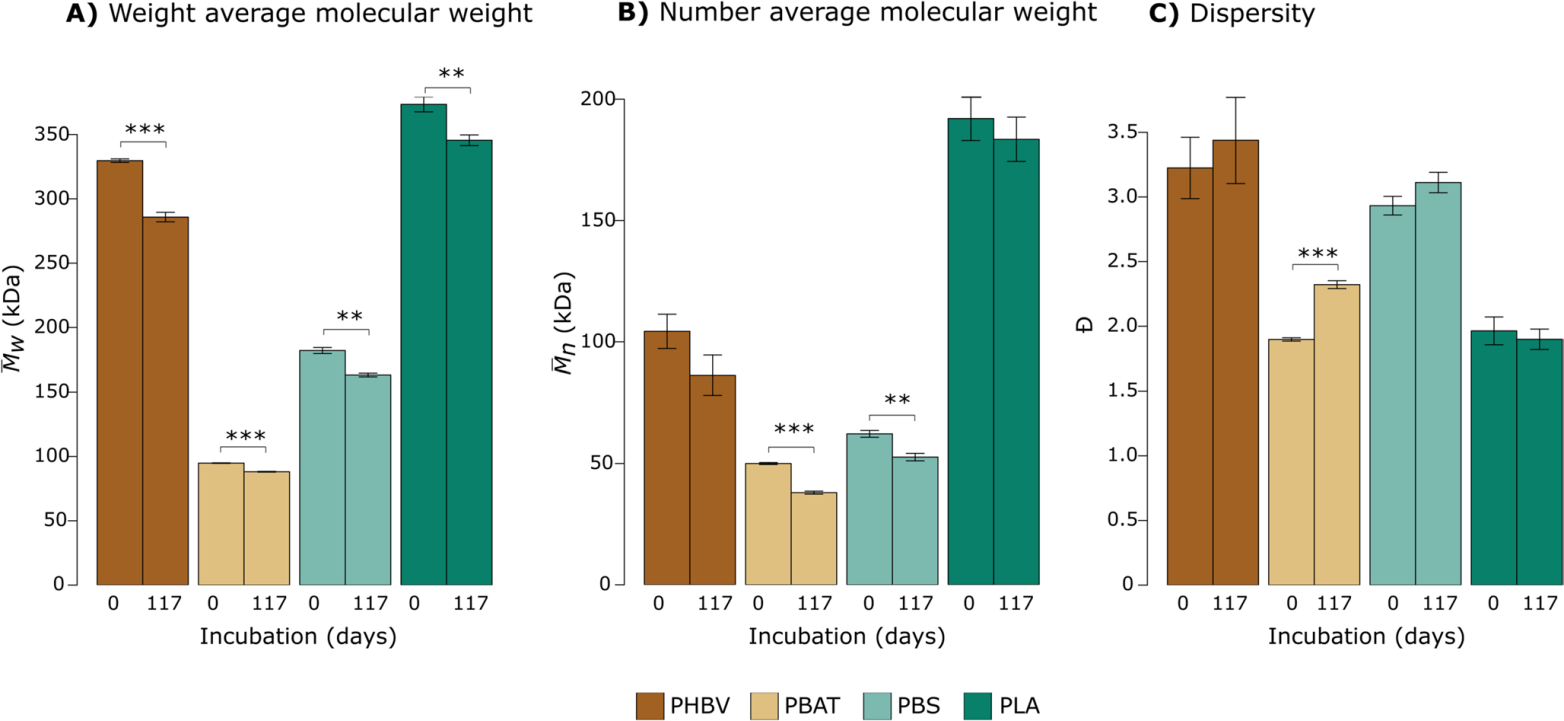
Physicochemical properties of non-buried (0 days) and buried (117 days) biodegradable polymer (BP) samples. Parameters measured were: (**A**) Weight average molecular weight ($\:{\stackrel{-}{M}}_{W}$); (**B**) Number average molecular weight ($\:{\stackrel{-}{M}}_{n}$) and (**C**) Dispersity (**Đ**). The days on which the samples were collected (0 or 117) are shown on the x axis. Error bars represent 95% confidence intervals of the means. Asterisks indicate that the difference between the 0- and 117-day values within a BP type (PHBV, PBAT, PBS or PLA) was significant using a student’s t-test where *p* < 0.001***; *p* < 0.01**; *p* < 0.05*

Significant reductions in $\:{\stackrel{-}{\:M}}_{W}$between buried and non-buried samples, indicating chain scission in the bulk of the BP, were observed for all samples (Fig. 3a), with BP type significantly impacting the degree of BP $\:{\stackrel{-}{\:M}}_{W}$loss and following the order: PHBV (14% loss) > PBS (10% loss) > PBAT (7% loss) and PLA (7% loss) over 117 days (Fig. S1a). PBAT and PBS, but not PHBV or PLA, showed significant reductions in $\:{\stackrel{-}{M}}_{n}$ following burial (Fig. 3b). PBAT showed the largest increase in dispersity following soil burial (Fig. S1) and was the only BP for which this change was significant (Fig. 3c).

### Bacterial and Fungal Biomass

**Fig. 4:**
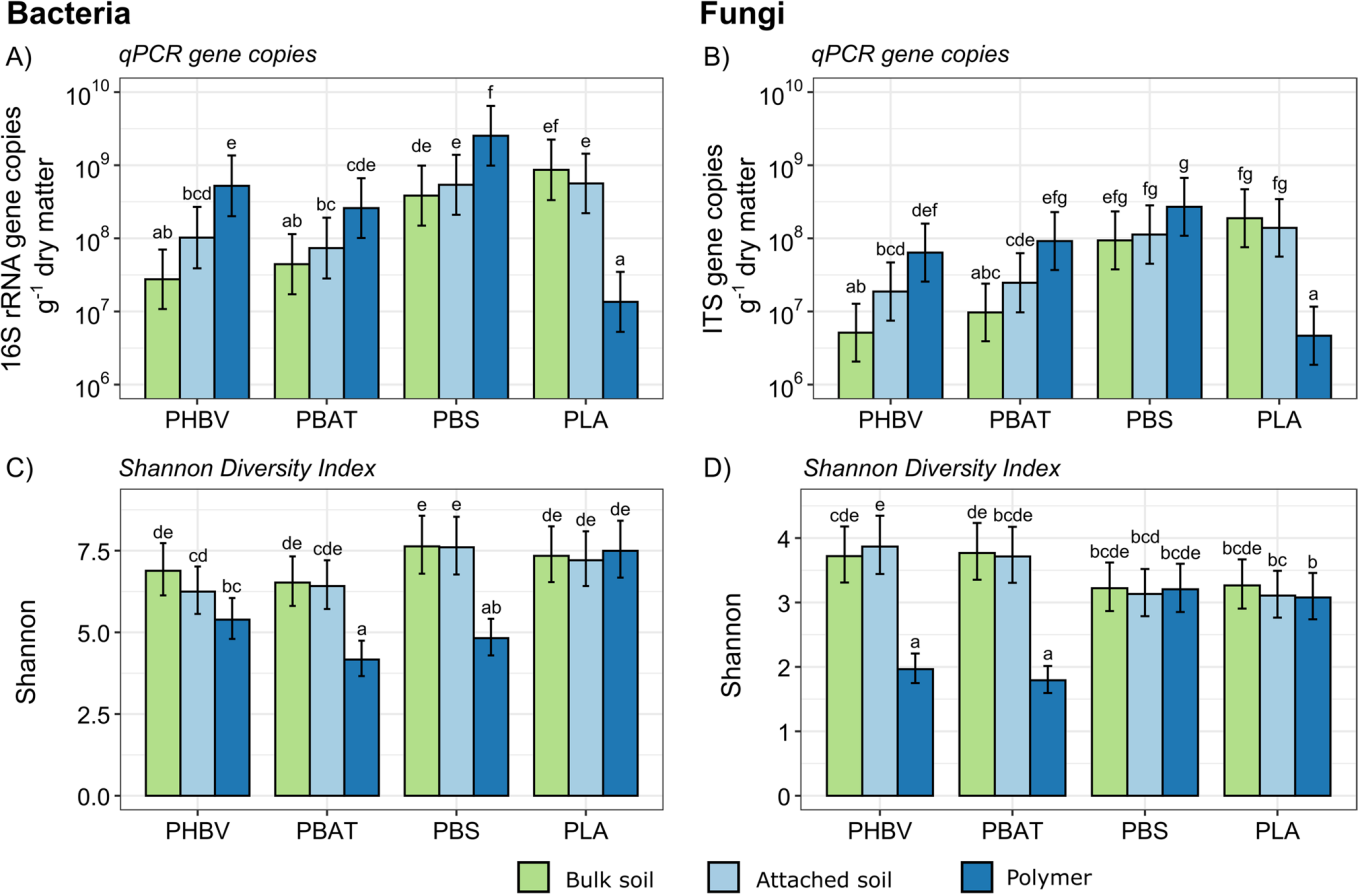
Bacterial and fungal biomass load and alpha diversity (Shannon’s Diversity Index) in polymer and soil compartments for each BP type. qPCR measurements of the number of copies of (**A**) bacterial or (**B**) fungal marker genes per gram of dry material (soil or polymer) and Shannon’s diversity index of (**C**) bacterial and (**D**) fungal observed taxonomic units (OTUs) were measured for each treatment. Data represent estimated marginal means ± 95% confidence intervals for each treatment combination. Letters above the bars represent the result of *post hoc* pairwise comparisons based on generalized linear mixed effects models testing the main and interactive effects of compartment and BP type on biomass or Shannon. *P*-values were adjusted for multiple comparisons using the Šidák method. Groups sharing the same letter are not significantly different at *p* < 0.05

GLMM indicated a significant main effect of BP type, and a significant interactive effect of BP type and compartment on bacterial and fungal biomass load (Table S6). Biomass load generally increased in the polymer compartment relative to the bulk soil for PHBV, PBAT and PBS, with this being significant for PHBV and PBAT (bacterial and fungi) and PBS (bacteria) (Fig. 4a, b). In contrast, for PLA, the polymer had significantly lower bacterial and fungal biomass load than the surrounding soils (Fig. 4a, b), suggesting minimal microbial colonization of this BP. Similar results were observed when expressing biomass load per unit area (gene copies cm⁻²) instead of per gram of dry matter. For example, PLA had the lowest bacterial and fungal biomass per unit area, and PBS had the highest, exceeding PHBV for both 16 S and ITS, and PBAT for 16 S (Fig. S2). Additionally, more biomass was detected in bulk soils from PLA and PBS than from PBAT or PHBV (Fig. 4a, b).

### Alpha Diversity

GLMM indicated significant main and interactive effects of BP type and Compartment on all alpha diversity metrics (Table ; S_obs_, Chao1, Shannon, and PD). While the influence of BP type was significant, that of compartment was generally stronger (Table S6). PHBV and PBAT exhibited significant decreases in bacterial and fungal alpha diversity in the polymer relative to the bulk soil and/or attached soil compartments (Fig. 4c, d, S3). For PBS, the polymer exhibited a significant reduction in bacterial, but not fungal diversity relative to the surrounding soils (Fig. 4c, d, S3). In contrast, both bacterial and fungal alpha diversity was unchanged in the PLA plastisphere relative to the bulk soil (Fig. 4c, d, S3).

### Bacterial and Fungal Community Composition

**Fig. 5:**
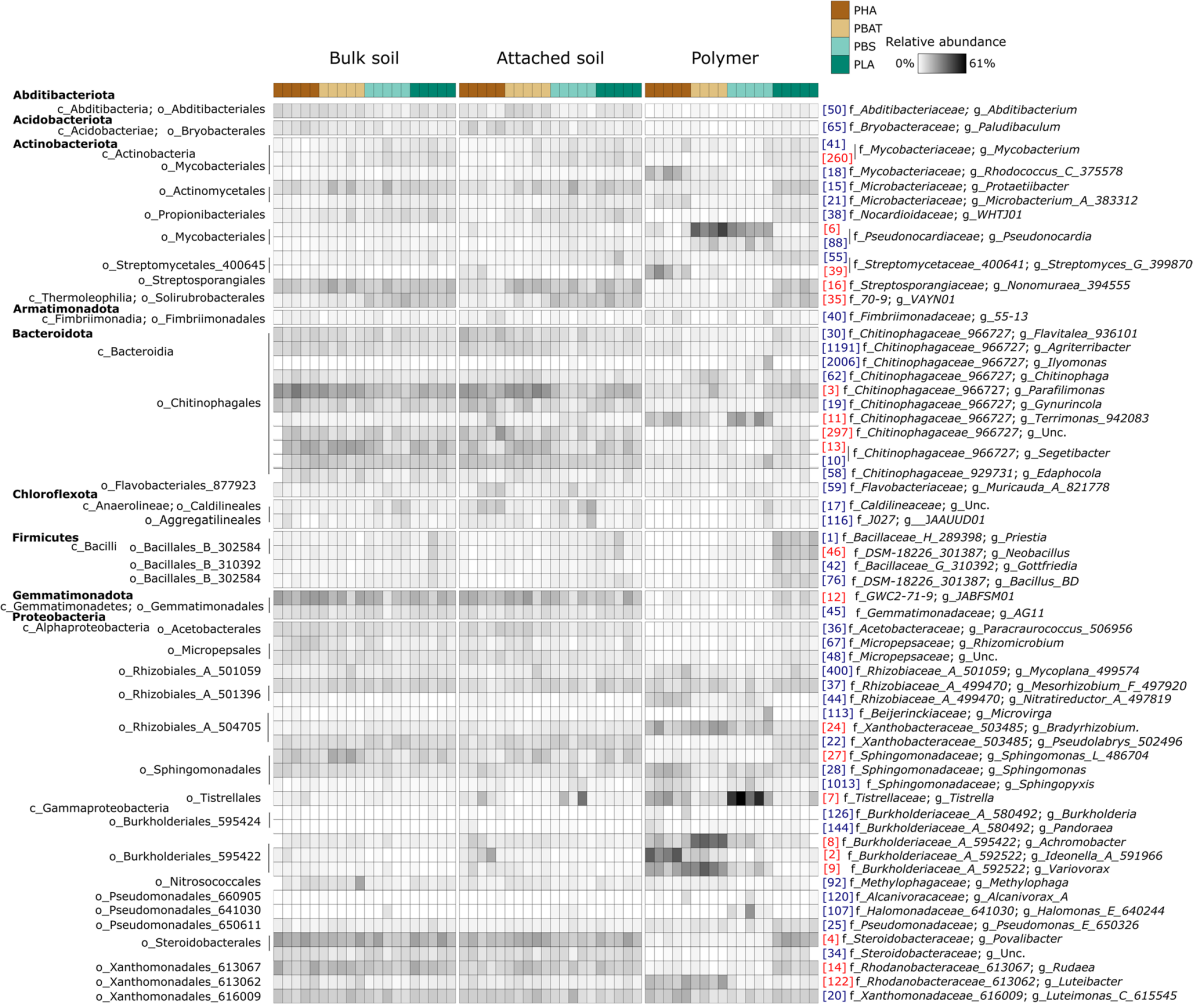
Heatmap showing the frequency of bacterial operational taxonomic units (OTUs) with ≥ 1% mean relative abundance in any treatment group. Relative abundances are Hellinger-transformed. OTU IDs (in brackets) are consistent across figures; red labelled OTUs correspond to those highlighted in the redundancy analysis (Fig. 7)

**Fig. 6:**
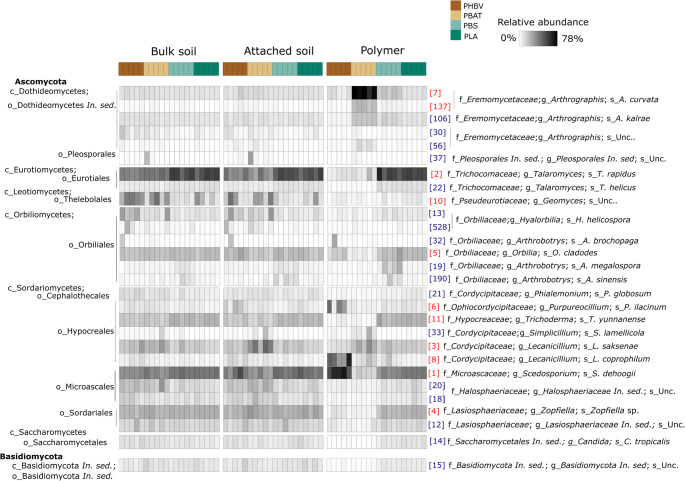
Heatmap showing the frequency of fungal operational taxonomic units (OTUs) with ≥ 1% mean relative abundance in any treatment group. Relative abundances are Hellinger-transformed. OTU IDs (in brackets) are consistent across figures; red labelled OTUs correspond to those highlighted in the redundancy analysis (Fig. 7)

The dominant bacterial phyla, viz. those present at ≥ 1% mean relative abundance within any treatment, comprised Abditibacteriota, Acidobacteriota, Actinobacteriota, Armatimonadota, Bacteroidota, Chloroflexicota, Firmicutes, Gemmatimonadota and Proteobacteria (Fig. 5). Fungal communities were dominated by members of the Ascomycota (comprising classes Dothideomycetes, Eurotiomycetes, Leotiomycetes, Orbiliomycetes, Sordariomycetes and Saccharomycetes) and Basidiomycota (Fig. 6). The composition of bacterial and fungal communities differed significantly between BP type and compartments (Table 1). For bacteria, the percent R^2^ values from the PERMANOVA model indicated that compartment had a slightly larger influence on the compositional variation between samples than BP type, while the opposite was observed for fungi (Table 1).

**Table 1:** Results from PERMANOVA models summarising the main and interactive effects of BP type (PHBV, PBAT, PBS, PLA), and compartment (bulk soil, attached soil, polymer) on the composition of bacterial and fungal communities

Target	Response variable	Predictor	F value	*R*^2^ (%)	*P* value	
Bacteria (16 S)	OTU relative abundance (Hellinger transformed)	BP type	10.13	21.30	< 0.001	***
		Compartment	19.82	27.70	< 0.001	***
		BP type: Compartment	4.33	18.20	< 0.001	***
	Weighted UniFrac differences	BP type	14.05	23.40	< 0.001	***
		Compartment	25.88	28.70	< 0.001	***
		BP type: Compartment	6.53	21.80	< 0.001	***
Fungi (ITS)	OTU relative abundance (Hellinger transformed)	BP type	23.35	31.90	< 0.001	***
		Compartment	20.07	18.30	< 0.001	***
		BP type: Compartment	10.23	28.00	< 0.001	***

Asterisks represent the significance of model terms where: *p* < 0.001***; *p* < 0.01**; *p* < 0.05*

**Fig. 7:**
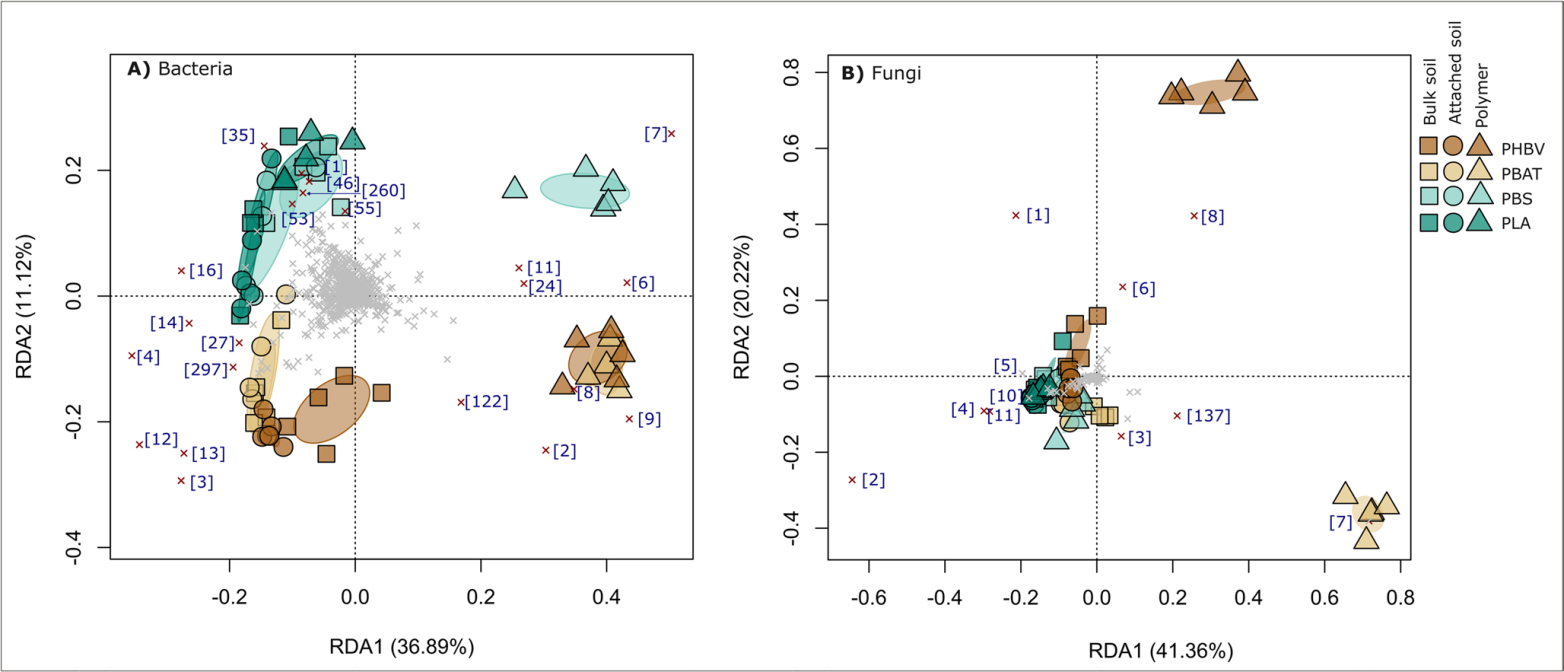
Redundancy analysis ordination constrained by BP type (PHBV, PBAT, PBS, PLA) and compartment (bulk soil, attached soil, polymer) highlighting differences in the composition of (**A**) bacterial or (**B**) fungal communities as represented by Hellinger transformed operational taxonomic unit (OTU) relative abundances. Ellipses represent standard deviations of the polymer group centroids. Crosses indicate operational taxonomic units (OTUs). Red crosses denote the highest discriminating OTUs on the RDA1 and RDA2 axes. The numbers in square brackets represent bacterial and fungal OTU IDs and are consistent with those in the corresponding heatmaps (Figs. 5 and 6). An enlarged view of the central region of the fungal ordination, providing greater resolution of PLA and PBAT community structure, is presented in Figure S4

Critically, bacterial communities associated with the PHBV, PBAT and PBS polymer compartments were highly distinct from those found in the soil compartments, whereas communities associated with the PLA polymer compartment clustered closely with the soil compartments, suggesting minimal compositional change for this BP type (Fig. 7).

Redundancy analysis highlighted that PHBV and PBAT polymer samples harboured similar bacterial communities and were positively correlated with the relative abundances of *Burkholderiaceae_A_592522* (OTUs 2, 8 and 9) and *Luteobacter* (OTU 122) populations (Figs. 5 and 7). The distinct bacterial composition of the PBS polymer, compared to that of PHBV and PBAT, was characterized by the increased relative abundance of *Tistrella* (OTU7) and *Terrimonas* (OTU11) populations (Figs. 5 and 7). *Pseudocardia* (OTU6) populations were positively correlated with the polymer compartments of both PBS and PBAT, suggesting some overlap in microbial colonisation between these BPs (Figs. 5 and 7). These correlations were also supported by indicator analyses (Table S7). The relative abundances of several Bacilli (OTUs 1, 42, 46, 72), Gemmatimonadales (OTUs 12, 45) and Steroidobacterales (OTUs 4, 34) decreased in the polymer compartments of PHBV, PBAT and PBS relative to the soils but were maintained on the PLA polymer relative to the soils (Figs. 5 and 7a).

For fungal communities, PBS and PLA samples showed minimal compositional differences between bulk soil, attached soil, and polymer compartments (Fig. 7b). However, closer inspection of the ordination revealed subtle distinctions between the polymer and soil compartments in PBS samples (Fig. S4). Conversely, fungal communities associated with the PHBV and PBAT polymer compartments differed markedly from those associated with the soil compartments, and in contrast to our observations for bacteria, differed from one another (Figs. 6 and 7). The PHBV polymer compartment was positively correlated with the relative abundances of *Lecanicillium coprophilum* (OTU8), *Scedosporium dehoogii* (OTU1) and to a lesser extent *Purpureocillium lilacinum* (OTU6) populations (Figs. 6 and 7b). The PBAT polymer compartment was heavily dominated by *Arthrographis curvata* populations (OTUs 7 and 137). These positive associations were also supported by indicator analyses (Table S8). Relative to the soil compartments, PHBV and PBAT polymers showed a reduction in *Talaromyces rapidus* (OTU2) and *Talaromyces helicus* (OTU22) populations (Figs. 5 and 7b).

Strong bacterial or fungal compositional shifts between the polymer and soil compartments were often accompanied by gradual shifts between the bulk and attached soils, but in general, the zone of influence of the BP was highly localised to the polymer compartment itself (Fig. 7).

## Discussion

Biodegradable polymers present a sustainable alternative to conventional plastics, however further research is needed to assess how well they fulfill this promise [20, 48]. Here, we used soil microbes as bioindicators to compare the impact of four commonly produced BPs – PHBV, PBAT, PBS and PLA – on the microbiome of the polymer itself, the attached soil and the bulk soil during soil burial. We also assessed indicators of BP degradation using a range of physicochemical, structural and visual metrics. Our findings characterize physicochemical and microbial responses associated with each BP type, providing insight into their environmental impact.

### Comparison of Physiochemical Changes between BP Types

All BP types showed signs of degradation after soil burial. However, the extent and manifestation of degradation, as assessed using visual, µXCT and GPC analyses, varied considerably across BP types. GPC measurements can be used as indicators of degradation by assessing the degree of chain scission in retrieved bulk samples through BP molecular weight and dispersity changes [49]. PHBV and PBS exhibited the largest $\:{\stackrel{-}{\:M}}_{W}$losses and pronounced visual evidence of edge erosion, whereas PBAT showed more extensive cracking and changes in dispersity. Patchy surface discoloration on PBS may reflect microbial colonization patterns influenced by surface heterogeneity, as fungi preferentially degrade amorphous regions of PBS [50]. While exhibiting a significant decrease in $\:{\stackrel{-}{\:M}}_{W}$following soil burial, in contrast to the other BP types, no visual evidence of degradation or change in porosity was observed for PLA after soil burial (Table S5). Our findings corroborate reports of the relatively slow degradation of PLA under ambient soil conditions [51, 52] as well as the slower PLA degradation compared to PHBV [53], PBAT [48, 54], and PBS [55, 56]. PLA degradation is known to be faster in thermophilic conditions relative to the mesophilic conditions used in this study due to the high glass transition temperature of PLA [57], which may partially account for our observations.

The increased dispersity and total porosity, in addition to the substantial surface cracking observed in PBAT relative to PHBV following soil burial is consistent with previous studies [58], and supports an internal-based degradation mechanism for PBAT. PHBV is thought to primarily follow a surface erosion mechanism [59] and this was supported by visible erosion being largely confined to the edge of the BP and the relatively small increase in open (but not closed) porosity in PHBV BP samples. The lack of change in dispersity in PHBV, PBS and PLA suggest that chain scission occurred randomly across the BP population, rather than selectively cleaving the longest chains, consistent with a non-selective, bulk hydrolytic degradation mechanism of polymer degradation [60].

### Differential Microbial Responses To Biodegradable Polymers

#### Biomass load

Relative to bulk soil, PHBV, PBAT and PBS polymer samples tended to have more bacterial and fungal load per gram, whereas PLA surfaces had less (Fig. 4), mirroring the presence or absence of surface roughness, cracking, and discolouration following soil burial (Fig. 2). These features likely represent microscale niches that facilitate microbial colonisation and substrate utilisation [21]. The greater biomass load detected in the PHBV, PBAT and PBS polymer compartments relative to soil compartments may be due to localised carbon sources generated during BP degradation [61]. Greater microbial growth rates observed in the PHBV plastisphere relative to the bulk soil of PHBV-treated soils suggest that PHBV has greater amounts of bioavailable carbon close to the polymer surface than in the surrounding soil [62], supporting our observations. On the other hand, Macan et al. (2025) reported that larger numbers of bacteria were able to be cultured from soil attached to plastic than from the plastic surfaces themselves [63]. These differences may be attributed to differences in plastic type, bacterial composition, or experimental practices, e.g., rinsed vs. scaped polymers and qPCR vs. culture-based quantification. Both qPCR and culture-based approaches for biomass quantification have limitations – culture-based methods capture only a subset of the microbial community, and normalization of gene copy numbers to dry weight can be difficult when comparing soils and biopolymers due to differences in their physicochemical properties as well as extraction and amplification efficiencies. Nevertheless, qPCR provides a practical means of estimating microbial abundance across heterogeneous substrates.

The observed decrease in ${\overline M}_W$in PLA BP samples after soil burial, combined with the higher water vapor transmission rate (WVTR) of PLA relative to PHBV [64], suggests that initial internal depolymerization may occur via abiotic hydrolytic scission induced by moisture uptake [65–67]. This initial hydrolysis of PLA, along with its relatively high WVTR, may be expected to promote microbial colonization by increasing surface moisture and generating localized zones of higher humidity. These conditions can facilitate microbial attachment, biofilm formation, and the subsequent utilization of hydrolysis products as carbon sources. However, the lower microbial biomass observed on PLA polymers compared to surrounding soil suggests that these substrates were either unavailable to the resident microbial community or that the diversity and abundance of organisms capable of utilizing them was limited in the standardized soil, a hypothesis supported by the limited changes to microbial alpha or beta diversity observed in the PLA polymer compartment. Given that PLA undergoes abiotic hydrolysis considerably more slowly than microbial enzymatic degradation [68], the limited PLA degradation observed in this study is consistent with the low microbial biomass detected on the polymer compartment.

Within the polymer compartment, PBS harboured the largest biomass load, followed by PHBV and PBAT, and PLA had the least biomass load (Fig. 4). This pattern was consistent irrespective of whether the gene copies were expressed per gram of dry matter or per unit area (Fig. S2). While PBS exhibited fewer signs of degradation, the fact that it was more porous than other BP types (Table S5) may have provided more microscale niches for microbial colonisation and explain why it supported the largest biomass [69]. Given the increased biomass detected on the PBS polymer sample, it is conceivable that given a longer incubation time, PBS may be the first BP to completely break down, in agreement with our hypothesis, and as reported previously [10]. For PBAT, non-uniform degradation has been reported and is attributed to its heterogeneous chemical structure [70]. This could lead to the formation of surface cracks and pores, likely facilitating microbial ingress by increasing the internal surface area [71], which has been shown to be positively correlated with the rate of biodegradation [72, 73]. PHBV appeared to degrade more uniformly, potentially enabling more consistent points of access into the polymer for microbial colonization.

Soils treated with PBAT, PHAs, and PLA microplastics have shown increases in biomass although results vary between studies. We could not determine whether biomass increased in bulk soil over the course of the experiment; however, by day 117, bulk soils associated with PBS and PLA exhibited higher biomass than those associated with PHBV and PBAT. This observation is interesting given the lack of colonisation on the PLA polymer itself. One hypothesis is that changes induced by the early stages of PLA degradation, such as acidification of the plastisphere [74] may have inhibited the colonisation of many bacterial and fungal microorganisms directly on the polymer surface, while allowing their proliferation further away where these changes were less pronounced. Our findings contrast with a recent study reporting that PBAT microplastics increased soil microbial biomass, whereas PLA microplastics had no effect [75].

#### Diversity

The increased microbial biomass load, decreased alpha diversity and changed beta diversity on PHBV, PBAT and PBS polymer compartments relative to the surrounding soils suggests the selective enrichment of specific taxa on the polymer compartments, consistent with general observations for soil plastispheres [61] and supporting our hypothesis of stronger community shifts near the polymer compartment. Conversely, PLA exhibited minimal visual signs of degradation, with microbial populations showing relatively little response to its presence, aligning with some studies [75] and contrasting with others [76, 77]. PLA degrading taxa are often thermophilic [78], however, taxa capable of degrading PLA have been recovered from ambient soil environments [79–81], indicating the potential for microbial colonisation and activity under these conditions.

PHBV and PBAT polymer compartments shared enriched bacterial assemblages including *Burkholderiaceae_A_592522* populations (OTUs 2, 8, 9), known for their association with and degradation of PHBV and PBAT BPs [82–85]. The PBS polymer compartment exhibited a distinct bacterial community structure to the polymer compartments of the other BPs, possibly indicating alternative degradation pathways to PHBV and PBAT. It was enriched with a *Terrimonas* (OTU 11) population, which contain plant growth promoting traits and microplastic tolerance [86], and a *Tistrella* (OTU 7) population, which is associated with petrochemical degradation [87, 88]. Class Bacilli (OTUs 1, 42, 46, 76), which modulate several key roles in the soil ecosystem were outcompeted in PHBV, PBAT and PBS polymers relative to the soils, but were not inhibited on PLA polymer compartments. *Bacillis* populations serve as PLA degraders in both meso- and thermophilic environments [79].

Regarding fungal communities, composition and alpha diversity were impacted on the polymer compartments of PHBV and PBAT, but PBS and PLA polymers were less impacted, suggesting a lack of fungal specialisation on these BPs. Similarly to our findings, members of the genus *Talaromyces*, which were markedly reduced on the polymer surfaces of PHBV and PBAT relative to the soil, were shown to be more highly associated with PBS than PHBV or PBAT during soil burial [18]. An enrichment of animal or plant pathogens have been reported in the fungal plastisphere [75, 89, 90], with the potential for this being more pronounced in biodegradable than non-biodegradable polymers [91]. Notably, *S. dehoogii*, which was enriched on the PHBV polymers, is one of the causal agents of scedosporiosis in humans [92]. *P. lilacium*, a known degrader of PHBV and PBAT [58, 93, 94], has antifungal properties and is used as a biocontrol agent against various plant pathogens [95, 96], while *L. coprophilum* is a mycoparasite [97]. This may contribute to their domination within the fungal plastispheres. *A. curvata*, which dominated the PBAT fungal plastisphere, produces large amounts of lipases [98], enzymes which degrade PBAT [99]. The localized changes observed in the PBHV, PBAT and PLA plastisphere communities could positively influence soil ecosystem functioning; for instance, enrichment of plant growth-promoting bacteria such as *Terrimonas* can enhance nutrient cycling and crop productivity. On the other hand, declines in microbial diversity can correlate with negative ecosystem effects [100].

The minimal enrichment of (i) bacterial and fungal (PLA) or (ii) fungal (PBS) communities on these BPs may be explained by a lack of taxa capable of degrading the substrates available from these BPs during the timeframe of the experiment. Microbes associated with plastispheres are ultimately determined by the indigenous communities present [101] and while PBAT and PHBV degrading taxa are abundant in natural soil environments [48, 102, 103], many known PLA-degraders were be found to be absent from industry standard soils [48]. Furthermore, relative to PHA -degraders, fewer PLA-colonising and degrading bacteria have been reported from soil environments [81, 103, 104]. Further studies may show whether PLA is inhospitable or actively inhibits microbial colonisation at the early stages of soil degradation. As environmental soils become more accustomed to PLA bioplastic waste, it is plausible that more PLA-degraders may accumulate or adapt in ambient soil environments [61]. Less is known regarding PBS degraders, but it has been proposed that fungal taxa capable of degrading PBS may also be limited in natural soils [50]. Nevertheless, a diverse community of BP-degrading microorganisms does not inherently ensure efficient degradation; instead, efficiency depends on the presence of key taxa, which could be few in number, with specialized biodegradative capabilities [7]. Indeed, although PBS polymers were not associated with decreased fungal diversity or large changes in fungal community composition, fungal biomass accumulated on the polymer, suggesting that PBS was colonized by fungi. Recently it was reported that PBS, in addition to PBAT, showed a marked change in fungal plastisphere diversity relative to the soil after 60 days burial, suggesting an enrichment of PBS fungal degraders at this early stage of degradation [105]. These differences may reflect variations in environmental or soil conditions, as well as differences in BP size.

We used bulk soil as a baseline for comparison with the polymer and attached soil compartments to explore polymer-induced microbial changes for each BP type. Interestingly, PBS and PLA bulk soils showed higher microbial biomass and some indicators of alpha diversity (16 S S_obs_, 16 S Chao1) than that of PHBV and PBAT. We applied randomized designs to account for any potential differences in starting community or incubation effects, however, is unclear whether these differences were pre-existing or BP–induced. Measuring soil parameters such as pH before and after incubation and including no-polymer controls could help clarify these findings and provide insight into potential underlying mechanisms. Standardized soil and pure BPs in microcosm setups were used in this study to provide controlled and reproducible conditions, thereby establishing a benchmark for comparison with other studies. However, this approach does not fully capture the complexity of natural field environments. Potential “jar effects,” such as oxygen limitation or CO₂ accumulation—conditions not typically present in open soil systems—may influence soil physicochemical properties. Moreover, microbial communities are highly variable among environmental soils [106]. In practice, BPs are often blended with other polymers or additives during manufacture, and these processes can also influence degradation and microbial communities [10, 25]. Therefore, follow-up studies conducted under field conditions with a range of plastic products will be essential to assess how these BPs respond to a broader range of biotic and abiotic factors. Microbial colonisation of BPs in soils is successional [107], so future studies tracking communities over time and space could also clarify these dynamics.

## Conclusions

Our findings emphasize the role of BP type in determining microbial disruption and physicochemical changes in soils. The observed changes in microbial diversity and biomass on the polymer compartments relative to the bulk soils suggests an ecological shift favouring specialised microorganisms in the PHBV, PBAT and PBS, but not PLA plastispheres in soil in the early stages of degradation. The reduced microbial colonisation of PLA in soil underscores the limitations of current biodegradable plastics in soils and highlights the need for informed BP selection to enhance environmental sustainability.

## Supplementary Information

Below is the link to the electronic supplementary material.

Supplementary Material 1 (PDF 661 KB)

## Data Availability

The raw reads described in this study are available at NCBI under BioProject: [PRJNA1237422](https:/www.ncbi.nlm.nih.gov/bioproject/PRJNA1237422).

## Declarations

### Data Statement

The raw reads described in this study are available at NCBI under BioProject: PRJNA1237422.
