# Supplementary material for: The Diversity of Plastisphere Bacterial and Fungal Communities Differs between Biodegradable Polymer Types in Soil
Source: Microb Ecol. 2026 Feb 3;89(1):56. doi: 10.1007/s00248-025-02677-z (PMC12913324; doi:10.1007/s00248-025-02677-z)
Supplement: Supplementary file 1 — Supplementary Material 1 (PDF 661 KB) [file 248_2025_2677_MOESM1_ESM.pdf]

Supplementary Information

The diversity of plastisphere bacterial and fungal communities differs between biodegradable polymer types in soil

Rebecca Lyons[a], Clement M. Chan[b], Catherine M. E. Hodal[a], Andrew R. Parry[a], Paul Lant[b], Steven Pratt[b], Bronwyn Laycock[b], Paul G. Dennis[a*]

[a]*School of the Environment, The University of Queensland, St Lucia, QLD, Australia;* [b]*School of Chemical Engineering, The University of Queensland, St Lucia, QLD, Australia;* *Corresponding author: p.dennis@uq.edu.au

Content:

Table S1 Material used to prepare polymer sheets……………………………………..…..3

Table S2 Barrel and die temperature profiles……………………………….…....……4

Table S3 Primer and gBlock sequences…………………………………….………....5

Table S4 Assumption tests for statistical models…………………………………..…6

Table S5 Porosity measurements of buried and non-buried BP pieces……………......7

Table S6 Generalised linear mixed effects models assessing the main and interactive effects of BP type and compartment on alpha diversity and biomass…………………8

Table S7 Bacterial operational taxonomic unit indicator analyses……………………..9

Table S8 Fungal operational taxonomic unit indicator analyses………………..……..13

Fig. S1 Percentage change in physicochemical properties of buried (117 days) relative to non-buried (0 days) BP pieces……………..………………………..……………………14

Fig. S2 Bacterial and fungal biomass (qPCR) on polymer compartments……………15

Fig. S3 Bacterial and fungal alpha diversity in bulk soil, attached soil and polymer compartments for PHBV, PBAT, PBS and PLA……………………...........................16

Fig. S4 High resolution distance-based redundancy analysis ordinations illustrating differences in fungal community composition……………………………..................17

Supplementary methods……………….………………………………………………......18

Supplementary references………………………………………………………..…….23

Table S1 Materials used to prepare biodegradable polymer sheets. $\bar{M}_n$: Number average molecular weight; $\bar{M}_W$: Weight average molecular weight; Đ: Dispersity index.

**Parameter**	Poly(3-hydroxybutyrate-co-3-hydroxyvalerate)	Poly(lactic acid)	Poly(butylene adipate-co-terephthalate)	Poly(butylene succinate)
**Acronym**	PHBV	PLA	PBAT	PBS
**Form**	Powder	Pellet	Pellet	Pellet
**Additives**	1 mol% 3-hydroxyvalerate (3HV)			
**Trade name**	ENMAT Y1000	2003D	EcoFlex C1200	PBE003
**Supplier**	TianAn Biopolymer, China	Natureworks, U.S.A.	BASF, Germany	NaturePlast, France
$\bar{M}_n$	192	232	41	64
$\bar{M}_W$	455	414	94	180
**Đ**	2.3	1.8	2.3	2.8

Table S2 Individual barrel and die temperature profile used for all biodegradable polymer samples

	Temperature (°C)
Zone 1 (Feed)	100
Zone 2	180
Zone 3	180
Zone 4	180
Zone 5	180
Zone 6	180
Zone 7	180
Zone 8	165
Zone 9	160
Die	160

Table S3. Primers and gBLOCK sequences used in this study. The unique 8bp molecular identifier (MID) sequences are represented by Ns.

Assay	Description	Name	Sequence	Target	Ref.
Bacterial amplicon sequencing	Primer	iTAG_1392wR	GTCTCGTGGGCTCGGAGATGTGTATAAGAGACAGACGGGCGGTGWGTRC	V6-V8 regions of 16S rRNA	[1]
	Primer	iTAG_MID_926F	TCGTCGGCAGCGTCAGATGTGTATAAGAGACAGNNNNNNNNAAACTYAAAKGAATTGRCGG		[1]
Fungal amplicon sequencing	Primer	iTAG_ITS4R	GTCTCGTGGGCTCGGAGATGTGTATAAGAGACAGTCCTCCGCTTATTGATATGC	Fungal ITS2	[2]
	Primer	gITS7F	TCGTCGGCAGCGTCAGATGTGTATAAGAGACAGANNNNNNNNGTGARTCATCGARTCTTTG		[3]
Bacterial qPCR	Primer	338-Eub	CTCCTACGGGAGGCAGCACT	16S rRNA	4]
	Primer	513-Eub	ATTACCGCGGCTGCTGG		[5]
	gBLOCK	*Escherichia coli* gBLOCK	CAGCCACACTGGAACTGAGACACGGTCCAGACTCCTACGGGAGGCAGCACTGGGGAATATTGCACATTGGGCGCAAGCCTGATGCAGCCATGCCGCGTGTATGAAGAAGGCCTTCGGGTAGTAAAGTACTTTCAGCGGGGAGGAAGGGAGTAAAGTTAATACCTTAGCTCATTGACGTTACCCGCAGAAGAAGCACCGGCTAACTCCGTGCCAGCAGCCGCGGTAATACGGAGGGTGCAAGCGTTAATCGGAATTACTGGGCGTAAAGCGCACGCAGGCGGTTTGTTAAGTCAGATGTGA	*E. coli* strain IF 20-12 (NCBI ref: MW044632.1), position 300-600	[6]
Fungal qPCR	Primer	ITS1f	CTTGGTCATTTAGAGGAAGTAA	Fungal ITS1	[7]
	Primer	5.8 s	CGCTGCGTTCTTCATCG		[8]
	gBLOCK	*Fusarium oxysporum* gBLOCK	TGGCTAAGTCTAGTCGTAACCTTGGTCATTTAGAGGAAGTAAAAGTCGTAACAAGGTCTCCGTTGGTGAACCAGCGGAGGGATCATTACCGAGTTATACAACTCATCAACCCTGTGAACATACCTAAAACGTTGCTTCGGCGGGAACAGACGGCCCTGTAACAACGGGCCGCCCCCGCCAGAGGACCCCTAACTCTGTTTTTATAATGTTTTTCTGAGTAAACAAGCAAATAAATTAAAACTTTCAACAACGGATCTCTTGGCTCTGGCATCGATGAAGAACGCAGCGAAATGCGATAAGTAATGTGA	*F.oxysporum* isolate B40 (NCBI ref: MN567689.1) position 21-308.	[6]

Table S4. Results of Shapiro-Wilk (normality of residuals) and Levene's (homogeneity of variance) tests used to assess the assumptions of linear mixed-effects models (LMMs) and one-way analysis of variance (ANOVA) models. LMMs were applied with 'BP type' and 'Compartment' as fixed effects and 'Jar' as a random effect.

Response variable	Model	Non-transformed		Log10-transformed	
		Shapiro-Wilk	Levene's	Shapiro-Wilk	Levene's
Bacteria					
Predicted OTUs (Chao1)	LMM	0.001 ***	0.837	0.011	0.550
Observed OTUs	LMM	0.007	0.566	<0.001 ***	0.258
Phylogenetic Diversity	LMM	0.010 **	0.662	0.081	0.219
Shannon's Diversity Index	LMM	<0.001 ***	0.012 *	<0.001 ***	0.002 **
qPCR (gene copies g^{-1} dry matter)	LMM	<0.001 ***	<0.001 ***	0.039 *	0.741
qPCR (gene copies cm^{-2}; polymer only)	ANOVA	0.099	0.009	0.248	0.886
Fungi					
Predicted OTUs (Chao1)	LMM	<0.001 ***	0.476	0.002 **	0.825
Observed OTUs	LMM	0.712	0.505	0.623	0.566
Shannon's Diversity Index	LMM	0.084	0.575	<0.001 ***	0.012 *
qPCR (gene copies g^{-1} dry matter)	LMM	<0.001 ***	0.155	0.261	0.566
qPCR (gene copies cm^{-2}; polymer only)	ANOVA	<0.001 ***	0.205	0.126	0.955
Physiochemical changes					
% Mn change	ANOVA	0.934	0.106		
% Mw change	ANOVA	0.274	0.625		
% Dispersity change	ANOVA	0.186	0.0779		

Table S5. Total, open, and closed porosity measurements of non-buried (0 days) or buried (117 days) BP (PHBV, PBAT, PBS or PLA) derived from image processing of X-ray micro computed tomography images in three-dimensional space

**Biopolymer type**	**Total porosity (vol. %)**			**Open porosity (vol. %)**			**Closed porosity (vol. %)**		
	0 days	117 days	Net change	0 days	117 days	Net change	0 days	117 days	Net change
**PHBV**	0.50	1.28	+0.78	0.47	1.25	+0.79	0.029	0.031	+0.002
**PBAT**	0.38	1.70	+1.32	0.30	1.17	+0.83	0.041	0.54	+0.50
**PBS**	1.03	0.97	-0.06	0.71	0.77	+0.06	0.320	0.22	-0.10
**PLA**	0.37	0.41	+0.04	0.35	0.39	+0.04	0.022	0.017	-0.005

Table S6. Results from generalised linear mixed effects models (GLMM) with a gamma distribution and log link function summarising the main and interactive effects of BP type (PHBV, PBAT, PBS, PLA) and compartment (bulk soil, attached soil, polymer) on the biomass and alpha diversity of bacterial and fungal communities. 'Compartment' and 'BP type' were included as fixed effects and 'jar' was included as a random effect. Asterisks represent the significance of model terms where: $p < 0.001$***; $p < 0.01$**; $p < 0.05$*.

**Response variable**	**Predictor variable**	χ^2	***P* value**	
Bacteria				
	BP type	79.4	<0.001	***
Predicted OTUs (Chao1)	Compartment	99.4	<0.001	***
	BP type : Compartment	15.8	<0.001	***
	BP type	45.7	<0.001	***
Observed OTUs	Compartment	140.4	<0.001	***
	BP type : Compartment	111.0	<0.001	***
	BP type	26.7	0.000	***
Phylogenetic Diversity	Compartment	144.1	<0.001	***
	BP type : Compartment	113.5	<0.001	***
	BP type	17.6	0.033	*
Shannon's Diversity Index	Compartment	45.8	<0.001	***
	BP type : Compartment	9.1	<0.001	***
	BP type	55.1	<0.001	***
Biomass (qPCR)	Compartment	30.2	<0.001	***
	BP type: Compartment	138.2	<0.001	***
Fungi				
	BP type	8.6	0.035	*
Predicted OTUs (Chao1)	Compartment	90.6	<0.001	***
	BP type : Compartment	71.4	<0.001	***
	BP type	10.2	0.017	*
Observed OTUs	Compartment	223.6	<0.001	***
	BP type : Compartment	203.1	<0.001	***
	BP type	8.765	0.033	*
Shannon's Diversity Index	Compartment	67.206	0.000	***
	BP type : Compartment	64.306	0.000	***
	BP type	12.465	0.006	**
Biomass (qPCR)	Compartment	216.663	0.000	***
	BP type: Compartment	190.046	0.000	***

Table S7 Bacterial operational taxonomic units (OTUs) indicator analyses.

OTU	BP type	Compartment	*P* value		Phylum	Class	Order	Family	Genus
Otu67	PHBV	Bulk soil	0.006	**	Proteobacteria	Alphaproteobacteria	Micropepsales	Micropepsaceae	*Rhizomicrobium*
Otu103	PHBV	Attached soil	0.013	*	Bacteroidota	Bacteroidia	Chitinophagales	Chitinophagaceae_966727	*VBAT01*
Otu157	PHBV	Attached soil	0.036	*	Acidobacteriota	Acidobacteriae	Bryobacterales	Bryobacteraceae	*Fen-178*
Otu165	PHBV	Attached soil	0.023	*	Acidobacteriota	Blastocatellia	Pyrinomonadales	Pyrinomonadaceae_426821	*OLB17_426821*
Otu19	PHBV	Attached soil	0.019	*	Bacteroidota	Bacteroidia	Chitinophagales	Chitinophagaceae_966727	*Gynurincola*
Otu214	PHBV	Attached soil	0.002	**	Acidobacteriota	Acidobacteriae	Bryobacterales	Bryobacteraceae	*Palsa-89*
Otu2295	PHBV	Attached soil	0.003	**	Gemmatimonadota	Gemmatimonadetes	Gemmatimonadales	GWC2-71-9	*JABFSM01*
Otu291	PHBV	Attached soil	0.006	**	Verrucomicrobiota	Verrucomicrobiae	Opitutales	Opitutaceae	*ER46*
Otu30	PHBV	Attached soil	0.013	*	Bacteroidota	Bacteroidia	Chitinophagales	Chitinophagaceae_966727	*Flavitalea_936101*
Otu3036	PHBV	Attached soil	0.023	*	Bacteroidota	Bacteroidia	Cytophagales	Cyclobacteriaceae_900466	*Chryseotalea*
Otu3221	PHBV	Attached soil	0.01	*	Bacteroidota	Bacteroidia	Cytophagales	Cyclobacteriaceae_900466	*Chryseotalea*
Otu326	PHBV	Attached soil	0.017	*	Gemmatimonadota	Gemmatimonadetes	Gemmatimonadales	Gemmatimonadaceae	*AG11*
Otu59	PHBV	Attached soil	0.013	*	Bacteroidota	Bacteroidia	Flavobacteriales_877923	Flavobacteriaceae	*Muricauda_A_821778*
Otu64	PHBV	Attached soil	0.003	**	Desulfobacterota_D	UBA1144	UBA2774	UBA2774	*CR02bin9*
Otu65	PHBV	Attached soil	0.002	**	Acidobacteriota	Acidobacteriae	Bryobacterales	Bryobacteraceae	*Paludibaculum*
Otu73	PHBV	Attached soil	0.015	*	Bacteroidota	Bacteroidia	Cytophagales	Cyclobacteriaceae_900466	*Chryseotalea*
Otu1013	PHBV	Polymer	0.009	**	Proteobacteria	Alphaproteobacteria	Sphingomonadales	Sphingomonadaceae	*Sphingopyxis*
Otu118	PHBV	Polymer	0.038	*	Bacteroidota	Bacteroidia	Sphingobacteriales	Sphingobacteriaceae	*Parapedobacter*
Otu129	PHBV	Polymer	0.011	*	Proteobacteria	Gammaproteobacteria	Burkholderiales_592522	Burkholderiaceae_A_592522	*Alicycliphilus*
Otu149	PHBV	Polymer	0.033	*	Bacteroidota	Bacteroidia	Bacteroidales	Tenuifilaceae	*SB25*
Otu18	PHBV	Polymer	0.002	**	Actinobacteriota	Actinomycetia	Mycobacteriales	Mycobacteriaceae	*Rhodococcus_C_375578*
Otu191	PHBV	Polymer	0.027	*	Planctomycetota	Planctomycetia	Isosphaerales	Isosphaeraceae	*Singulisphaera*
Otu197	PHBV	Polymer	0.02	*	Actinobacteriota	Actinomycetia	Actinomycetales	Cellulomonadaceae	*Cellulosimicrobium*
Otu2	PHBV	Polymer	0.01	*	Proteobacteria	Gammaproteobacteria	Burkholderiales_592522	Burkholderiaceae_A_592522	*Ideonella_A_591966*
Otu21	PHBV	Polymer	0.001	**	Actinobacteriota	Actinomycetia	Actinomycetales	Microbacteriaceae	*Microbacterium_A_383312*
Otu2106	PHBV	Polymer	0.013	*	Proteobacteria	Alphaproteobacteria	Sphingomonadales	Sphingomonadaceae	*Allosphingosinicella*
Otu2310	PHBV	Polymer	0.036	*	Bacteroidota	Bacteroidia	Chitinophagales	Chitinophagaceae_966727	*Flavitalea_936101*

Otu255	PHBV	Polymer	0.005	**	Myxococcota_A_473307	Polyangia_463783	Nannocystales	Nannocystaceae	*Nannocystis*
Otu28	PHBV	Polymer	0.001	**	Proteobacteria	Alphaproteobacteria	Sphingomonadales	Sphingomonadaceae	*Sphingopyxis*
Otu39	PHBV	Polymer	0.016	*	Actinobacteriota	Actinomycetia	Streptomycetales_400645	Streptomycetaceae_400641	*Streptomyces_G_399870*
Otu400	PHBV	Polymer	0.016	*	Proteobacteria	Alphaproteobacteria	Rhizobiales_A_501059	Rhizobiaceae_A_501059	*Mycoplana_499574*
Otu44	PHBV	Polymer	0.008	**	Proteobacteria	Alphaproteobacteria	Rhizobiales_A_501396	Rhizobiaceae_A_499470	*Nitratireductor_A_497819*
Otu588	PHBV	Polymer	0.034	*	Firmicutes_D	Bacilli	Paenibacillales	NBRC-103111	*Paenibacillus_V*
Otu80	PHBV	Polymer	0.047	*	Proteobacteria	Alphaproteobacteria	Sphingomonadales	Sphingomonadaceae	*Sphingomonas_L_486704*
Otu81	PHBV	Polymer	0.004	**	Actinobacteriota	Actinomycetia	Streptomycetales_400645	Streptomycetaceae_400641	*Streptomyces_G_399870*
Otu887	PHBV	Polymer	0.001	**	Bacteroidota	Bacteroidia	Chitinophagales	Chitinophagaceae_966727	*Pseudobacter*
Otu945	PHBV	Polymer	0.012	*	Proteobacteria	Gammaproteobacteria	Burkholderiales_592524	Burkholderiaceae_A_574908	*Paucimonas*
Otu95	PHBV	Polymer	0.011	*	Chloroflexota	Chloroflexia	Thermomicrobiales	Unc.	Unc.
Otu13	PBAT	Bulk soil	0.003	**	Bacteroidota	Bacteroidia	Chitinophagales	Chitinophagaceae_966727	*Segetibacter*
Otu182	PBAT	Bulk soil	0.039	*	Proteobacteria	Alphaproteobacteria	Sphingomonadales	Sphingomonadaceae	*Erythrobacter_484322*
Otu27	PBAT	Bulk soil	0.004	**	Proteobacteria	Alphaproteobacteria	Sphingomonadales	Sphingomonadaceae	*Sphingomonas_L_486704*
Otu390	PBAT	Bulk soil	0.026	*	Proteobacteria	Gammaproteobacteria	Pseudomonadales_660905	Alcanivoracaceae	*Alcanivorax_A*
Otu92	PBAT	Bulk soil	0.002	**	Proteobacteria	Gammaproteobacteria	Nitrosococcales	Methylophagaceae	*Methylophaga*
Otu1191	PBAT	Attached soil	0.022	*	Bacteroidota	Bacteroidia	Chitinophagales	Chitinophagaceae_966727	*Agriterribacter*
Otu154	PBAT	Attached soil	0.026	*	Proteobacteria	Alphaproteobacteria	Dongiales	Dongiaceae	*Dongia_507335*
Otu190	PBAT	Attached soil	0.013	*	Verrucomicrobiota	Verrucomicrobiae	Opitutales	Opitutaceae	*ER46*
Otu3	PBAT	Attached soil	0.007	**	Bacteroidota	Bacteroidia	Chitinophagales	Chitinophagaceae_966727	*Parafilimonas*
Otu349	PBAT	Attached soil	0.043	*	Verrucomicrobiota	Verrucomicrobiae	Pedosphaerales	UBA11358	*UBA11358*
Otu36	PBAT	Attached soil	0.005	**	Proteobacteria	Alphaproteobacteria	Acetobacterales	Acetobacteraceae	*Paracraurococcus_506956*
Otu50	PBAT	Attached soil	0.003	**	Armatimonadota	Abditibacteria	Abditibacteriales	Abditibacteriaceae	*Abditibacterium*
Otu122	PBAT	Polymer	0.009	**	Proteobacteria	Gammaproteobacteria	Xanthomonadales_613062	Rhodanobacteraceae_613062	*Luteibacter*
Otu24	PBAT	Polymer	0.003	**	Proteobacteria	Alphaproteobacteria	Rhizobiales_A_504705	Xanthobacteraceae_503485	*Bradyrhizobium*
Otu2985	PBAT	Polymer	0.002	**	Proteobacteria	Gammaproteobacteria	Burkholderiales_595422	Burkholderiaceae_A_595422	*Pigmentiphaga*
Otu301	PBAT	Polymer	0.001	**	Proteobacteria	Alphaproteobacteria	Rhizobiales_A_500472	Rhizobiaceae_A_500472	*Allorhizobium*
Otu344	PBAT	Polymer	0.003	**	Proteobacteria	Gammaproteobacteria	Burkholderiales_595422	Burkholderiaceae_A_595422	*Bordetella_C*
Otu3523	PBAT	Polymer	0.006	**	Proteobacteria	Gammaproteobacteria	Burkholderiales_592522	Burkholderiaceae_A_592522	*Hydrogenophaga_590395*
Otu6	PBAT	Polymer	0.001	**	Actinobacteriota	Actinomycetia	Mycobacteriales	Pseudonocardiaceae	*Pseudonocardia*

Otu62	PBAT	Polymer	0.02	*	Bacteroidota	Bacteroidia	Chitinophagales	Chitinophagaceae_966727	*Chitinophaga*
Otu8	PBAT	Polymer	0.001	**	Proteobacteria	Gammaproteobacteria	Burkholderiales_595422	Burkholderiaceae_A_595422	*Achromobacter*
Otu9	PBAT	Polymer	0.001	**	Proteobacteria	Gammaproteobacteria	Burkholderiales_592522	Burkholderiaceae_A_592522	*Variovorax*
Otu102	PBS	Attached soil	0.007	**	Acidobacteriota	Thermoanaerobaculia	UBA5704	UBA5704	Unc.
Otu112	PBS	Attached soil	0.033	*	Myxococcota_A_473307	Polyangia_463783	Polyangiales	Polyangiaceae	Unc.
Otu116	PBS	Attached soil	0.037	*	Chloroflexota	Anaerolineae	Aggregatilineales	J027	*JAAUUD01*
Otu148	PBS	Attached soil	0.009	**	Chloroflexota	Anaerolineae	Anaerolineales	EnvOPS12	*OLB14*
Otu162	PBS	Attached soil	0.001	**	Proteobacteria	Alphaproteobacteria	Rhizobiales_A_504723	Aestuariivirgaceae	*Aestuariivirga*
Otu200	PBS	Attached soil	0.012	*	Proteobacteria	Alphaproteobacteria	Rhizobiales_A_504705	Xanthobacteraceae_503485	*Pseudorhodoplanes*
Otu57	PBS	Attached soil	0.019	*	Planctomycetota	Planctomycetia	Planctomycetales	UBA10511	*UBA10511*
Otu96	PBS	Attached soil	0.024	*	Acidobacteriota	Vicinamibacteria	Vicinamibacterales	SCN-69-37	*SCN-69-37*
Otu107	PBS	Polymer	0.019	*	Proteobacteria	Gammaproteobacteria	Pseudomonadales_641030	Halomonadaceae_641030	*Halomonas_E_640244*
Otu11	PBS	Polymer	0.003	**	Bacteroidota	Bacteroidia	Chitinophagales	Chitinophagaceae_966727	*Terrimonas_942083*
Otu113	PBS	Polymer	0.001	**	Proteobacteria	Alphaproteobacteria	Rhizobiales_A_504705	Beijerinckiaceae	*Microvirga*
Otu167	PBS	Polymer	0.029	*	Armatimonadota	Abditibacteria	Abditibacteriales	Abditibacteriaceae	*Abditibacterium*
Otu173	PBS	Polymer	0.003	**	Proteobacteria	Gammaproteobacteria	Burkholderiales_597432	Rhodocyclaceae	*Azoarcus_F_597129*
Otu1992	PBS	Polymer	0.011	*	Proteobacteria	Alphaproteobacteria	Rhodobacterales	Rhodobacteraceae	*Haematobacter*
Otu2006	PBS	Polymer	0.004	**	Bacteroidota	Bacteroidia	Chitinophagales	Chitinophagaceae_966727	*Ilyomonas*
Otu258	PBS	Polymer	0.005	**	Proteobacteria	Alphaproteobacteria	Rhizobiales_A_504705	Xanthobacteraceae_503485	*Tardiphaga*
Otu323	PBS	Polymer	0.005	**	Proteobacteria	Alphaproteobacteria	Rhodobacterales	Rhodobacteraceae	*Amaricoccus*
Otu3493	PBS	Polymer	0.014	*	Bacteroidota	Bacteroidia	Cytophagales	Cyclobacteriaceae_900466	*UBA2336*
Otu361	PBS	Polymer	0.009	**	Proteobacteria	Alphaproteobacteria	Rhizobiales_A_504705	Xanthobacteraceae_503485	*GCF-000702305*
Otu386	PBS	Polymer	0.021	*	Proteobacteria	Alphaproteobacteria	Unc.	Unc.	Unc.
Otu429	PBS	Polymer	0.001	**	Proteobacteria	Alphaproteobacteria	Rhodobacterales	Rhodobacteraceae	*Paracoccus*
Otu447	PBS	Polymer	0.006	**	Actinobacteriota	Actinomycetia	Actinomycetales	Microbacteriaceae	*Leucobacter*
Otu7	PBS	Polymer	0.001	**	Proteobacteria	Alphaproteobacteria	Tistrellales	Tistrellaceae	*Tistrella*
Otu123	PLA	Bulk soil	0.023	*	Actinobacteriota	Actinomycetia	Mycobacteriales	Mycobacteriaceae	*Nocardia*
Otu171	PLA	Bulk soil	0.039	*	Planctomycetota	Planctomycetia	Pirellulales	Thermoguttaceae	*Thermogutta*
Otu42	PLA	Bulk soil	0.019	*	Firmicutes_D	Bacilli	Bacillales_B_310392	Bacillaceae_G_310392	*Gottfriedia*
Otu90	PLA	Bulk soil	0.008	**	Proteobacteria	Alphaproteobacteria	Rhizobiales_A_502138	Devosiaceae	Unc.

Otu106	PLA	Attached soil	0.048	*	Proteobacteria	Gammaproteobacteria	Methylococcales	Methylothermaceae	Unc.
Otu139	PLA	Attached soil	0.034	*	Chlamydiota	Chlamydiia	Chlamydiales_778124	Parachlamydiaceae	*Protochlamydia*
Otu142	PLA	Attached soil	0.008	**	Actinobacteriota	Actinomycetia	Mycobacteriales	Mycobacteriaceae	*Mycobacterium*
Otu153	PLA	Attached soil	0.023	*	Actinobacteriota	Actinomycetia	Actinomycetales	Microbacteriaceae	*Microbacterium_A_383299*
Otu186	PLA	Attached soil	0.016	*	Planctomycetota	Planctomycetia	Pan216	Pan216	*Pan216*
Otu210	PLA	Attached soil	0.005	**	Proteobacteria	Alphaproteobacteria	Rhizobiales_A_504705	Beijerinckiaceae	*Methylocystis*
Otu260	PLA	Attached soil	0.025	*	Actinobacteriota	Actinomycetia	Mycobacteriales	Mycobacteriaceae	*Mycobacterium*
Otu2848	PLA	Attached soil	0.018	*	Actinobacteriota	Actinomycetia	Mycobacteriales	Mycobacteriaceae	*Mycobacterium*
Otu38	PLA	Attached soil	0.042	*	Actinobacteriota	Actinomycetia	Propionibacteriales	Nocardioidaceae	*WHTJ01*
Otu53	PLA	Attached soil	0.028	*	Actinobacteriota	Thermoleophilia	Solirubrobacterales	Unc.	Unc.
Otu56	PLA	Attached soil	0.003	**	Actinobacteriota	Actinomycetia	Propionibacteriales	Nocardioidaceae	*Nocardioides_A_392796*
Otu87	PLA	Attached soil	0.033	*	Proteobacteria	Alphaproteobacteria	Rhizobiales_A_504705	Beijerinckiaceae	*Methylocella*

Table S8 Fungal operational taxonomic units (OTUs) indicator analyses

OTU	BP type	Compartment	*P* value		Phylum	Class	Order	Family	Genus_species
Otu21	PHBV	Bulk soil	0.002	**	Ascomycota	Sordariomycetes	Cephalothecales	Cephalothecaceae	*Phialemonium_globosum*
Otu10	PHBV	Bulk soil	0.002	**	Ascomycota	Leotiomycetes	Thelebolales	Pseudeurotiaceae	*Geomyces_sp*
Otu30	PHBV	Bulk soil	0.004	**	Ascomycota	Dothideomycetes	Dothideomycetes_ord_Incertae_sedis	Eremomycetaceae	*Arthrographis_sp*
Otu13	PHBV	Bulk soil	0.036	*	Ascomycota	Orbiliomycetes	Orbiliales	Orbiliaceae	*Hyalorbilia_helicospora*
Otu1	PHBV	Polymer	0.001	**	Ascomycota	Sordariomycetes	Microascales	Microascaceae	*Scedosporium_dehoogii*
Otu8	PHBV	Polymer	0.001	**	Ascomycota	Sordariomycetes	Hypocreales	Cordycipitaceae	*Lecanicillium_coprophilum*
Otu6	PHBV	Polymer	0.004	**	Ascomycota	Sordariomycetes	Hypocreales	Ophiocordycipitaceae	*Purpureocillium_lilacinum*
Otu3	PBAT	Attached soil	0.001	**	Ascomycota	Sordariomycetes	Hypocreales	Cordycipitaceae	*Lecanicillium_saksenae*
Otu35	PBAT	Attached soil	0.004	**	Ascomycota	Sordariomycetes	Microascales	Halosphaeriaceae	*Halosphaeriaceae_sp*
Otu27	PBAT	Attached soil	0.006	**	Ascomycota	Sordariomycetes	Cephalothecales	Cephalothecaceae	*Phialemonium_globosum*
Otu18	PBAT	Attached soil	0.006	**	Ascomycota	Sordariomycetes	Microascales	Halosphaeriaceae	*Halosphaeriaceae_sp*
Otu20	PBAT	Attached soil	0.008	**	Ascomycota	Sordariomycetes	Microascales	Halosphaeriaceae	*Halosphaeriaceae_sp*
Otu26	PBAT	Attached soil	0.021	*	Ascomycota	Sordariomycetes	Sordariales	Lasiosphaeriaceae	*Zopfiella_sp*
Otu56	PBAT	Polymer	0.001	**	Ascomycota	Dothideomycetes	Dothideomycetes_ord_Incertae_sedis	Eremomycetaceae	*Arthrographis_sp*
Otu7	PBAT	Polymer	0.001	**	Ascomycota	Dothideomycetes	Dothideomycetes_ord_Incertae_sedis	Eremomycetaceae	*Arthrographis_curvata*
Otu137	PBAT	Polymer	0.001	**	Ascomycota	Dothideomycetes	Dothideomycetes_ord_Incertae_sedis	Eremomycetaceae	*Arthrographis_curvata*
Otu106	PBAT	Polymer	0.001	**	Ascomycota	Dothideomycetes	Dothideomycetes_ord_Incertae_sedis	Eremomycetaceae	*Arthrographis_kalrae*
Otu33	PBAT	Polymer	0.013	*	Ascomycota	Sordariomycetes	Hypocreales	Cordycipitaceae	*Simplicillium_lamellicola*
Otu190	PBS	Attached soil	0.014	*	Ascomycota	Orbiliomycetes	Orbiliales	Orbiliaceae	*Arthrobotrys_megalospora*
Otu19	PBS	Polymer	0.001	**	Ascomycota	Orbiliomycetes	Orbiliales	Orbiliaceae	*Arthrobotrys_megalospora*
Otu72	PLA	Attached soil	0.017	*	Ascomycota	Eurotiomycetes	Eurotiales	Aspergillaceae	*Penicillium_citrinum*
Otu29	PLA	Polymer	0.016	*	Ascomycota	Eurotiomycetes	Eurotiales	Aspergillaceae	*Aspergillus_insuetus*
Otu11	PLA	Polymer	0.026	*	Ascomycota	Sordariomycetes	Hypocreales	Hypocreaceae	*Trichoderma_yunnanense*
Otu22	PLA	Polymer	0.03	*	Ascomycota	Eurotiomycetes	Eurotiales	Trichocomaceae	*Talaromyces_helicus*

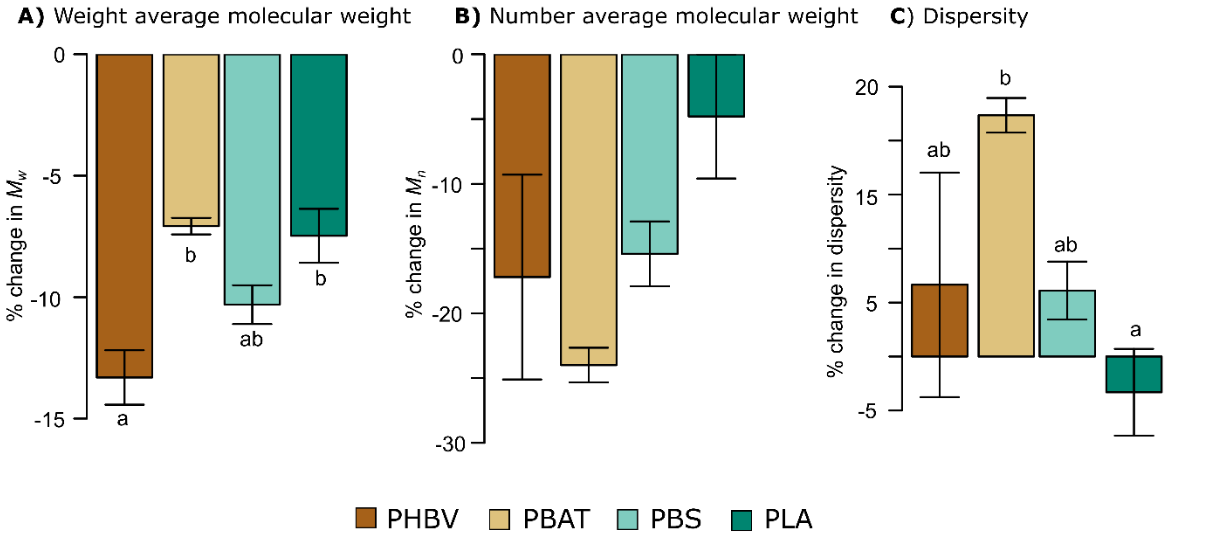

Fig. S1 Percentage change in physicochemical properties of buried (117 days) relative to non-buried (0 days) BP pieces. Parameters measured were (A) Weight average molecular weight ($\overline{M}_W$); (B) Number average molecular weight ($\overline{M}_n$); and (C) Dispersity index (Đ). Data shown are the mean and standard errors of the percentage change in five incubated BP pieces relative to the mean value of five non-incubated BP pieces. Letters above the graphs represent Tukey's *post-hoc* analyses applied to a one-way ANOVA examining the effect of BP type (PHBV, PBAT, PBS, PLA) on the variable ($\overline{M}_n$, $\overline{M}_W$, Đ) where treatments sharing the same letters are not significantly different (P>0.05).

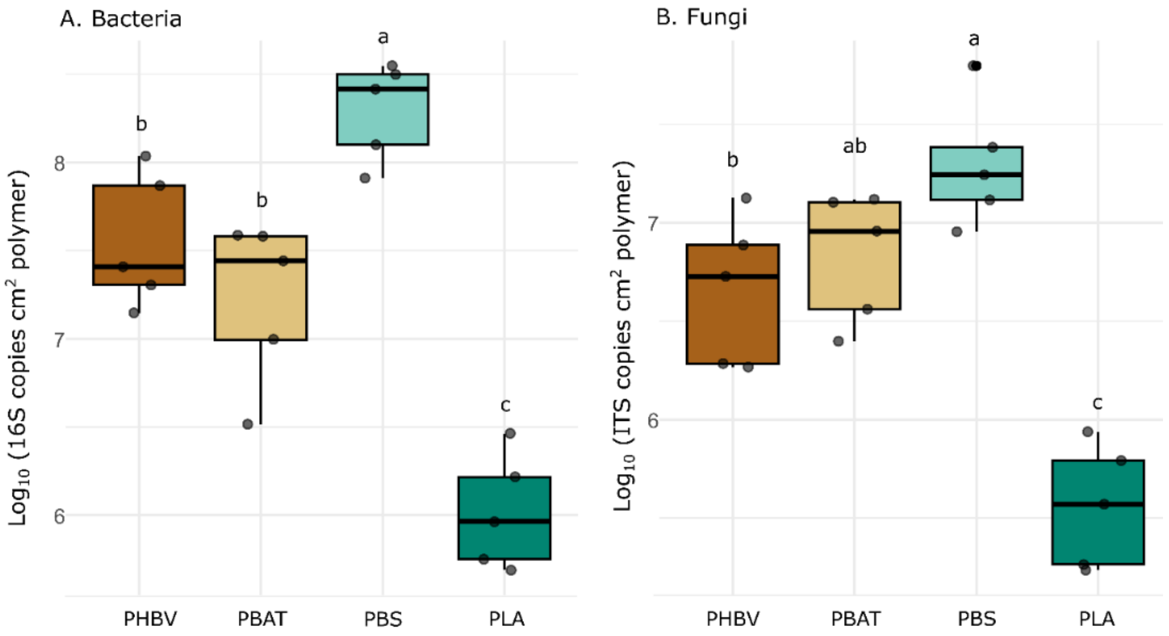

Fig. S2 Bacterial and fungal biomass load (as estimated using qPCR) on polymer compartments following soil burial. Boxplots show (A) 16S rRNA gene copies and (B) ITS copies per cm² of polymer surface for the four BP types. Each point represents an individual replicate. Different letters above the boxes indicate statistically significant differences between BP types ($p < 0.05$, ANOVA with *post-hoc* test).

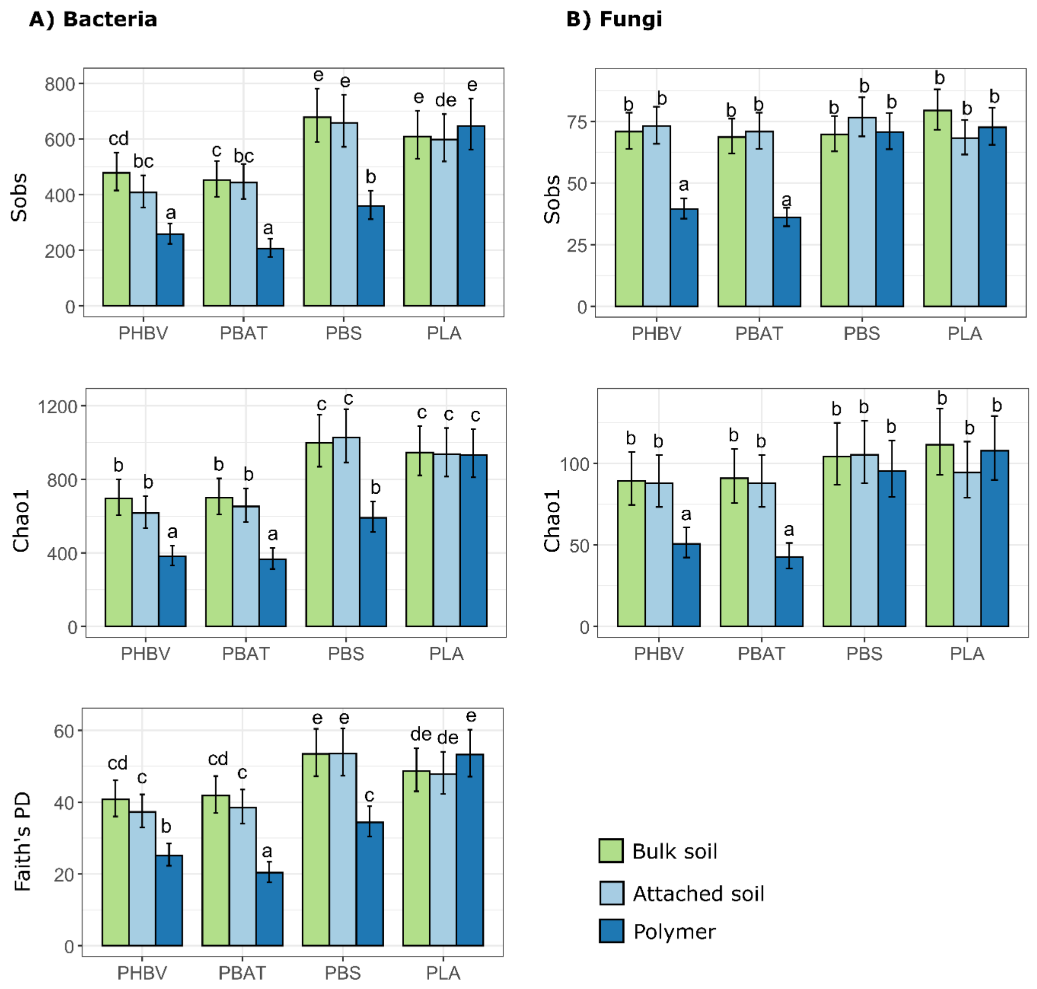

Fig. S3 Barplots representing the alpha diversity of bacterial and fungal communities associated with polymer and soil compartments for each BP type. Data shown are the estimated marginal means ±95% confidence intervals for: A) the observed numbers of bacterial OTUs (S_{obs}), predicted bacterial richness (Chao1) and Faith's phylogenetic diversity and B) fungal S_{obs} and Chao1, for each treatment combination. Letters above the bars represent the result of *post hoc* pairwise comparisons based on generalized linear mixed models testing the main and interactive effects of compartment (Bulk soil, attached soil, polymer) and BP type (PHBV, PBAT, PBS, PLA) on each alpha diversity metric. *P*-values were adjusted for multiple comparisons using the Šidák method. Groups sharing the same letter are not significantly different at $p < 0.05$.

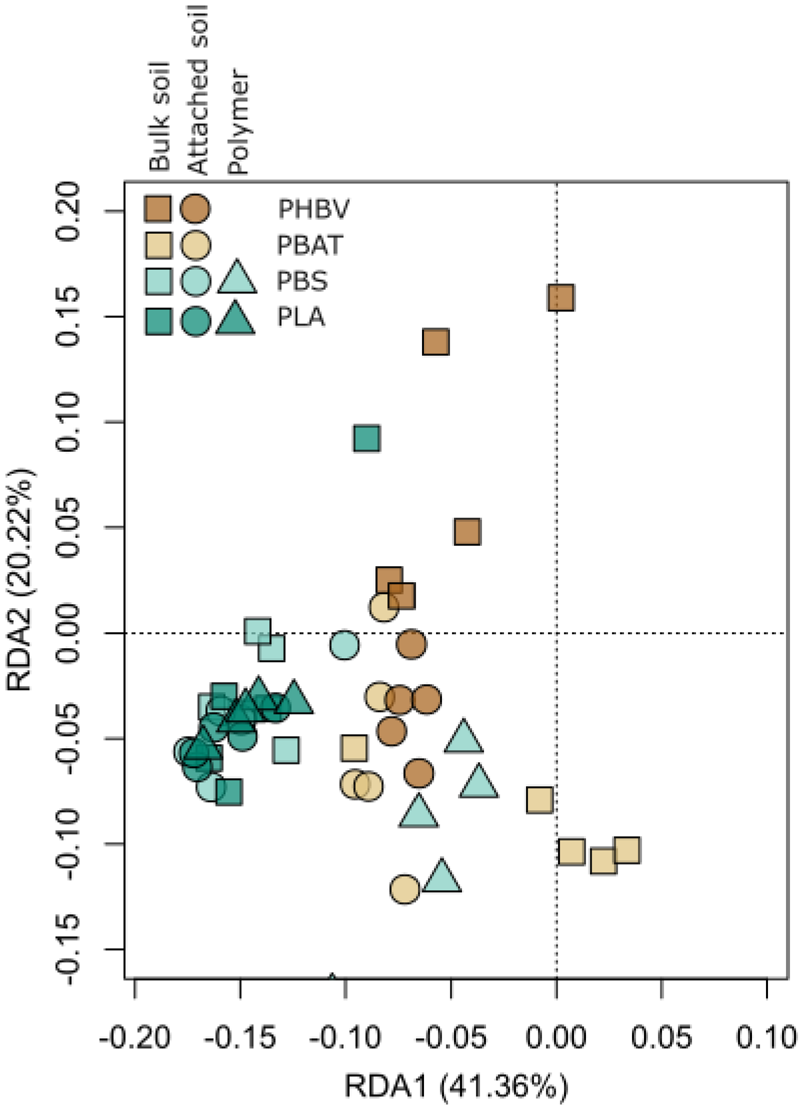

Fig. S4 Distance-based redundancy analysis (db-RDA) ordinations illustrating differences in fungal community composition between compartments for the four BP types (PHBV, PBAT, PBS, and PLA). The plot complements Fig. 7b by using constrained axes to better highlight differences among bulk soil (circles), attached soil (squares), and polymer (triangles) compartments within the PBS and PLA treatments. Polymer-associated samples from PHBV and PBAT fall outside the plotted axis range and are therefore not shown.

Supplementary methods

Micro X-ray computed tomography (µXCT)

µXCT was used to generate internal and external three-dimensional images of BP samples to measure changes in porosity and characterize physical cracks and erosion associated with degradation. High-resolution µXCT imaging was performed using The Lions Australia Skyscan 1272 desktop X-ray microscopy platform (version 1.1.19, Bruker, Belgium). A 3 mm x 10 mm specimen was cut from each BP sheet for imaging. The specimens were scanned at 50 kV and 200 µA with an exposure time of 1218 ms and 3 x 3 binning with an effective pixel size of 3 µm. A 0.25 mm aluminium filter was used with a frame averaging of 3 and 0.18° rotation step and 360° rotation around the sample.

The µXCT datasets were reconstructed using NRecon (Version 1.7.3.1, Bruker, Belgium) and InstaRecon (Version 2.0.4.6, InstaRecon, USA) using a Feldkamp algorithm. Beam hardening correction and ring artefact reduction were applied to each dataset. Three-dimensional images were constructed using CTvox (Version 3.3.0, Bruker, Belgium). Porosity content was estimated from 3D images using CTAn software (Version 1.19.11.1, Bruker, Belgium) by: 1) converting image slices to binary images via thresholding; 2) setting the boundary of the object, defined as the largest object stretched over holes with diameter of 20 pixels in 2D space; 3) calculating open porosity, defined as the pores connected to the boundary; and then 4) calculating closed porosity, defined as the porosity embedded within the object.

Molecular weight measurement

Gel permeation chromatography (GPC) was used to measure the weight average molecular weight ($\bar{M}_W$), the number average molecular weight ($\bar{M}_n$), and dispersity (Đ) of BP samples, as indicators of degradation. BP samples were dissolved at a concentration of 2.5 mg/mL in HPLC grade chloroform (LiChrosolv®, Merck 102444) in capped glass tubes on a heating block at 75ºC for 30 min. An Agilent 1260 Infinity Multi Detector Suite system (Cheshire, UK) fitted with a column set (3 x PLgel 10 µm MIXED-B columns in series) of molecular weight range from 0.5 to 1700 kDa was used. The columns were kept at 30ºC. A refractometer at 30ºC was used to detect the signals. A chloroform flow rate of 1 mL/min was maintained for the analysis. Narrowly distributed molecular weight polystyrene standards were used for calibration and the Mark-Houwink equation [9] was used for molecular weight calculations. The Mark-Houwink constants for polystyrene (K = 7.2 x 10^{-3} mL/g and α = 0.76) [10] , for P(3HB) (K = 7.7 x 10^{-3} mL/g and α = 0.82) [9], for PLA (K = 1.31 x 10^{-3} mL/g and α = 0.777) [11] , for PBAT (K = 1.29 x 10^{-3} mL/g and α = 0.87) [12] , and for PBS (K = 4.0 x 10^{-3} mL/g and α = 0.71) [13] in chloroform at 30°C were taken from the literature.

Quantitative real-time PCR

Standard curves comprised a 10-fold dilution series of each gBLOCK (Table S2) at 1 x 10^8 to 1x 10^1 gene copies. Each qPCR consisted of 2 µL template, 1x Power SYBR™ Green PCR Master Mix (Invitrogen), 1 µL of 5 ng/µl bovine serum albumin (BSA) and 0.1 µM (ITS) or 0.3 µM (16S) of each primer, made up to a total volume of 10 µL with water. No-template controls were included. Cycling conditions for the fungal qPCR were as follows: an initial denaturing step at 95 °C for 10 min followed

by 30 cycles of 95 °C for 15 s, 53°C s for 30 s (data acquisition) and 72°C for 30 s. The melt curve cycle was as follows: 95°C for 15 s, 60°C for 60 s and 95°C for 15 s. Cycling conditions for the bacterial qPCR were as above except annealing temp was 55°C and cycle number was 35. Reactions were carried out in triplicate using a Viia7 Instrument (ABI) and were quantified against the standard curves using QuantStudio™ Real-Time PCR Software, Version 1.2 (Applied Biosystems). Melting curves of the PCR products were evaluated to confirm that the fluorescence signal originated from specific PCR products and not from primer-dimers or amplification artifacts.

To convert qPCR data into marker gene copies g^{-1} dry material, the following calculation was performed:

$$Marker\ gene\ copies\ g^{-1}\ dry\ material = \left(\frac{Marker\ gene\ copies\ \mu L^{-1}}{g\ dry\ material\ \mu L^{-1}\ DNA\ extract}\right) * DNA\ dilution\ factor$$

To convert qPCR data into marker gene copies polymer area (cm-2), the following calculation was used. The total polymer area exposed to the soil for each replicate was calculated to be 2x faces (4.0 cm^2) + 4 x sides (0.4 cm^2), multiplied by two pieces of polymer = 19.2 cm^2. Each extraction from these polymer pieces was eluted in 50 µL.

$$Marker\ gene\ copies\ cm^{-2}\ polymer = \left(\frac{Marker\ gene\ copies\ \mu L^{-1}}{0.384\ cm^{-2}\ \mu L^{-1}}\right) * DNA\ dilution\ factor$$

Phylogenetic marker gene sequencing

A two-step PCR protocol was used to prepare 16S and ITS libraries. In PCR 1, universal primer pairs modified to contain a unique 8 bp molecular identifier (MID) and Illumina adapter for compatibility with the Nextera XT indices (Table S2) were used to amplify targets. In PCR 2, pools of up to 24 MID-barcoded amplicons combined in equimolar concentrations were subjected to dual indexing using the Nextera XT Index Kit (Illumina).

For 16S libraries, PCR 1 comprised 2.5 µL DNA template, 4 µL 5X Phire Green Reaction Buffer (Thermo Fisher), 0.4 µL of Phire Green Hot Start II DNA Polymerase (Thermo Fisher), 200 µM of each dNTP, and 250 nM of each primer made to a total volume of 20 µL with molecular biology grade water. Thermocycling conditions were as follows: 98°C for 45 sec; then 30 cycles of 98°C for 5 sec, 56°C for 5 sec, 72°C for 6 sec; followed by 72°C for 1 min. For ITS libraries, PCR 1 comprised 2.5 µL template DNA in 1x AmpliTaq Gold 360 master mix (Applied Biosystems) with 250 nM of each primer, made up to a total volume of 20 µL with water. Thermocycling conditions were as follows: 95 °C for 8 min; then 35 cycles of 95 °C for 20 s, 56 °C for 30 s, 72 °C for 45 sec min; followed by 72 °C for 7 min. Gel electrophoresis was used to confirm that no-template controls did not amplify products.

PCR 1 products were purified using an 18% suspension of Sera-Mag Speed-beads Carboxyl Magnetic Beads (GE Healthcare), added in a ratio of 1.8:1 vol PCR product and quantified using a Qubit according to the manufacturer's instructions. PCR 2 comprised 5 µL purified PCR1, 1x Phire Green Reaction Buffer (Thermo Fisher), 200 µM of each of the dNTPs (Invitrogen), 1 µL Phire Green Hot Start II DNA

Polymerase, and 1mM of each primer, made up to a total volume of 50 µL with water. Thermocycling conditions were as follows: 98 °C for 45 s; then 8 cycles of 95 °C for 5 s, 55 °C for 5 s, 72 °C for 6 s; followed by 72 °C for 1 min. Amplicons were then purified using an 18% suspension of Sera-Mag Speed-beads Carboxyl Magnetic Beads (GE Healthcare) as described above and pooled libraries were sequenced on an Illumina MiSeq.

Supplementary references

1. Engelbrektson A, Kunin V, Wrighton KC, et al (2010) Experimental factors affecting PCR-based estimates of microbial species richness and evenness. ISME J 4:642–647. https://doi.org/10.1038/ismej.2009.153
2. White TJ, Bruns TD, Lee SB, Taylor JW (1990) Amplification and direct sequencing of fungal ribosomal RNA Genes for phylogenetics. In: Innis MA, Gelfand DH, Sninsky JJ, White TJ (eds) PCR Protocols: A Guide to Methods and Applications. pp 315–322
3. Ihrmark K, Bödeker ITM, Cruz-Martinez K, et al (2012) New primers to amplify the fungal ITS2 region - evaluation by 454-sequencing of artificial and natural communities. FEMS Microbiol Ecol 82:666–677. https://doi.org/10.1111/j.1574-6941.2012.01437.x
4. Lane DJ (1991) 16S/23S rRNA Sequencing. In: Stackebrandt E, Goodfellow M (eds) Nucleic acid techniques in bacterial systematic. John Wiley and Sons, New York, pp 115–175
5. Muyzer G, de Waal EC, Uitterlinden AG (1993) Profiling of complex microbial populations by denaturing gradient gel electrophoresis analysis of polymerase chain reaction-amplified genes coding for 16S rRNA. Appl Environ Microbiol 59:695–700. https://doi.org/10.1128/aem.59.3.695-700.1993
6. Chan CM, Lyons R, Dennis PG, et al (2022) Effect of Toxic Phthalate-Based Plasticizer on the Biodegradability of Polyhydroxyalkanoate. Environ Sci Technol 56:17732–17742. https://doi.org/10.1021/acs.est.2c06583

7. Gardes M., Bruns TD (1993) ITS primers with enhanced specificity for basidiomycetes - application to the identification of mycorrhizae and rusts. Mol Ecol 2:113–118. https://doi.org/10.1111/j.1365-294X.1993.tb00005.x
8. Vilgalys R, Hester M (1990) Rapid genetic identification and mapping of enzymatically amplified ribosomal DNA from several Cryptococcus species. J Bacteriol 172:4238–4246. https://doi.org/10.1128/jb.172.8.4238-4246.1990
9. Marchessault RH, Okamura K, Su CJ (1970) Physical Properties of Poly(β-hydroxy butyrate). II. Conformational Aspects in Solution. Macromolecules 3:735–740. https://doi.org/10.1021/ma60018a005.
10. Wagner HL (1985) The Mark–Houwink–Sakurada Equation for the Viscosity of Atactic Polystyrene. J Phys Chem Ref Data 14:1101–1106. https://doi.org/10.1063/1.555740
11. Dorgan JR, Janzen J, Knauss DM, et al (2005) Fundamental solution and single-chain properties of polylactides. J Polym Sci B Polym Phys 43:3100–3111. https://doi.org/10.1002/polb.20577
12. Borman WFH (1978) Molecular weight–viscosity relationships for poly(1,4-butylene terephthalate). J Appl Polym Sci 22:2119–2126. https://doi.org/10.1002/app.1978.070220804
13. Garin M, Tighzert L, Vroman I, et al (2014) The influence of molar mass on rheological and dilute solution properties of poly(butylene succinate). J Appl Polym Sci 131:. https://doi.org/10.1002/app.40887
